# Genome-wide identification and characterization of the *GDP-L-galactose phosphorylase* gene family in bread wheat

**DOI:** 10.1186/s12870-019-2123-1

**Published:** 2019-11-26

**Authors:** Ronan C. Broad, Julien P. Bonneau, Jesse T. Beasley, Sally Roden, Joshua G. Philips, Ute Baumann, Roger P. Hellens, Alexander A. T. Johnson

**Affiliations:** 1 School of BioSciences, The University of Melbourne, Melbourne, Victoria 3010 Australia; 2 Centre for Tropical Crops and Biocommodities, Institute for Future Environments, Queensland University of Technology, Brisbane, Queensland 4001 Australia; 3 School of Agriculture, The University of Adelaide, Adelaide, South Australia 5064 Australia

**Keywords:** Ascorbic acid, Vitamin C, Upstream open reading frame, Phylogeny, Synteny, Gene expression, Transient expression

## Abstract

**Background:**

Ascorbate is a powerful antioxidant in plants and an essential micronutrient for humans. The *GDP-L-galactose phosphorylase* (*GGP*) gene encodes the rate-limiting enzyme of the L-galactose pathway—the dominant ascorbate biosynthetic pathway in plants—and is a promising gene candidate for increasing ascorbate in crops. In addition to transcriptional regulation, GGP production is regulated at the translational level through an upstream open reading frame (uORF) in the long 5′-untranslated region (5’UTR). The *GGP* genes have yet to be identified in bread wheat (*Triticum aestivum* L.), one of the most important food grain sources for humans.

**Results:**

Bread wheat chromosomal groups 4 and 5 were found to each contain three homoeologous *TaGGP* genes on the A, B, and D subgenomes (*TaGGP2-A*/*B*/*D* and *TaGGP1-A*/*B/D*, respectively) and a highly conserved uORF was present in the long 5’UTR of all six genes. Phylogenetic analyses demonstrated that the *TaGGP* genes separate into two distinct groups and identified a duplication event of the *GGP* gene in the ancestor of the *Brachypodium*/Triticeae lineage. A microsynteny analysis revealed that the *TaGGP1* and *TaGGP2* subchromosomal regions have no shared synteny suggesting that *TaGGP2* may have been duplicated via a transposable element. The two groups of *TaGGP* genes have distinct expression patterns with the *TaGGP1* homoeologs broadly expressed across different tissues and developmental stages and the *TaGGP2* homoeologs highly expressed in anthers. Transient transformation of the *TaGGP* coding sequences in *Nicotiana benthamiana* leaf tissue increased ascorbate concentrations more than five-fold, confirming their functional role in ascorbate biosynthesis *in planta*.

**Conclusions:**

We have identified six *TaGGP* genes in the bread wheat genome, each with a highly conserved uORF. Phylogenetic and microsynteny analyses highlight that a transposable element may have been responsible for the duplication and specialized expression of *GGP2* in anthers in the *Brachypodium*/Triticeae lineage. Transient transformation of the *TaGGP* coding sequences in *N. benthamiana* demonstrated their activity *in planta*. The six *TaGGP* genes and uORFs identified in this study provide a valuable genetic resource for increasing ascorbate concentrations in bread wheat.

## Background

Ascorbate, also known as vitamin C, is the most abundant water-soluble antioxidant in plant cells and plays important roles in photosynthetic function and stress tolerance [1, 2]. Ascorbate can mitigate the harmful effects of reactive oxygen species (ROS) produced by normal or stressed cellular metabolism, either directly as an ROS scavenger or indirectly as a substrate for the enzyme ascorbate peroxidase [3]. Increased biosynthesis of ascorbate in model and crop species has demonstrated that even minor increases in ascorbate can confer enhanced tolerance to a broad range of abiotic stresses [4, 5]. Additional roles for ascorbate within plant cells include: serving as an enzymatic cofactor [6] and precursor for organic acids [7], as well as influencing the cell cycle [8], cell wall expansion [9], and flowering time and senescence [10].

In humans, ascorbate is an essential micronutrient due to loss-of-function mutations within the *L-gulono-1,4-lactone oxidase* gene which encodes the enzyme responsible for catalysing the last step in vertebrate ascorbate biosynthesis [11]. Beyond its well-established role in preventing the disease scurvy, ascorbate plays key roles in many physiological processes important for human health, including promoting and regulating the uptake of dietary iron in the human digestive process [12] and serving as a cofactor for enzymes involved in epigenetic programming [5, 13]. Although ascorbate deficiency is considered a historical disease, suboptimal intake of ascorbate is currently present in both developing and developed countries [14], and the re-emergence of scurvy has been documented in Australia due to poor dietary habits [15].

Four pathways for ascorbate biosynthesis have been proposed in plants: the L-galactose, L-gulose, *myo*-inositol, and D-galacturonate pathways. Despite evidence for each of these pathways in plants, the L-galactose pathway, which converts D-fructose-6-P to ascorbate via eight enzymatic steps, is the dominant pathway leading to ascorbate biosynthesis in *Arabidopsis thaliana* [16–18], tomato (*Solanum lycopersicum* L.) [19], rice (*Oryza sativa* L.) [20], and green algae (*Chlamydomonas reinhardtii*) [21]. The final gene to be identified in the L-galactose pathway, *GDP-L-galactose phosphorylase* (*GGP*, also known as *VTC2*), was discovered and functionally characterized in *Arabidopsis* in 2007 [16, 22, 23]. The GGP enzyme catalyzes the conversion of GDP-L-galactose to L-galactose-1-P and represents the first committed step toward ascorbate biosynthesis. Several studies have now identified the GGP enzyme as the rate-limiting step in the L-galactose pathway [22, 24–26] and as the key regulatory enzyme in ascorbate biosynthesis [27]. Overexpression of *GGP* genes has increased ascorbate concentrations by up to 4.2-fold in *Arabidopsis* [24, 26], 3.1-fold in potato (*Solanum tuberosum* L.) [28], 2.1-fold in strawberry (*Fragaria x ananassa*) [28], 6.2-fold in tomato [28, 29], 1.4-fold in tobacco (*Nicotiana tabacum* L.) [30], and 2.6-fold in rice [25, 31].

A highly conserved upstream open reading frame (uORF)—a class of small ORFs located upstream of protein-coding major ORFs (mORFs) in the 5′-untranslated region (5’UTR) of mRNAs—of the *GGP* gene has been shown to regulate *GGP* translation and ascorbate concentrations [32–35]. Although uORFs are abundant in plant genomes with up to 60% of genes estimated to contain an uORF, less than 1% are conserved between plant species [36, 37]. The translational regulation of mORFs by highly conserved uORFs in response to cellular metabolite levels has been documented in several plant studies. For example, uORFs have been shown to regulate translation of the *Arabidopsis S-adenosylmethionine decarboxylase* and *polyamine oxidase 2* genes in repsonse to polyamines [38–41], the *Arabidopsis* S1 class *basic-leucine zipper* and tomato *ornithine decarboxylase* genes in response to sucrose [42–44], the *Arabidopsis phosphoethanolamine N-methyltransferase* gene in response to phosphocholine [45], and the *Arabidopsis heat shock trancription factor B1* gene in response to galactinol [46]. In the case of the *GGP* uORF, the encoded peptide which is predicted to initiate from a non-canonical AUC or ACG start-codon is proposed to cause ribosomal stalling, thereby preventing translation of the downstream *GGP* mORF under high ascorbate concentrations, whereas under low ascorbate concentrations the uORF is skipped and the *GGP* mORF translated [33]. The precise mechanism of how ascorbate may influence ribosomal initiation at the uORF or mORF has yet to be elucidated. Disruption of the *GGP* uORF through mutation of key residues was shown to interfer with ascorbate feedback regulation of translation and increase ascorbate concentration when transiently transformed in *Nicotiana benthamiana* [33]. Recently, CRISPR/Cas9 genome editing has been used to disrupt the *GGP* uORF in *Arabidopsis*, lettuce (*Lactuca sativa*), and tomato to increase ascorbate concentrations and enhance oxidative stress tolerance [34, 35].

Despite the importance of the *GGP* gene in ascorbate biosynthesis and regulation, characterisation of *GGP* genes within graminaceous species has been limited to rice and maize (*Zea mays* L.). Both rice and maize have one copy of the *GGP* gene present in their genomes, located on chromosomes Os11 and Zm6, respectively. Complete knock-out of the *OsGGP* gene in rice severely reduced foliar ascorbate concentrations by 80% and was correlated with reduced photosynthetic efficiency, lower biomass production, and reduced tolerance to ozone stress and zinc deficiency [20]. In maize, the *ZmGGP* gene is expressed during endosperm development and expression levels have been shown to be genotype-dependent [47].

Wheat is the world’s most cultivated crop in terms of land area and accounts for one fifth of the calories consumed by humans [48]. Abiotic stresses such as drought, salinity, and high temperature represent the major limiting factors in global wheat productivity [49]. Increased ascorbate concentrations in wheat could help to mitigate yield losses associated with these abiotic stresses. Like many other cereals, wheat contains negligible levels of ascorbate in the mature grain [50], therefore, ascorbate enrichment of wheat grain could also provide a novel means of improving human dietary intakes of this essential vitamin.

Here we expand upon our knowledge of *GGP* genes in graminaceous species by describing the *TaGGP* gene family in bread wheat. We describe the chromosomal location, confirm the presence of the highly conserved uORFs, present phylogenetic relationship, gene synteny, and tissue and developmental expression patterns, and demonstrate activity of the *TaGGP* coding sequences by transient transformation in *N. benthamiana*.

## Results

### Six *TaGGP* genes were identified on bread wheat chromosomal groups 4 and 5

A total of six *TaGGP* genes were identified within the bread wheat genome and their genomic locations were determined. Chromosomal groups 4 and 5 each contain three homoeologous *TaGGP* genes located on the A, B, and D subgenomes (*TaGGP2-A*/*TaGGP2-B*/*TaGGP2-D* and *TaGGP1-A*/*TaGGP1-B*/*TaGGP1-D*, respectively). All six genes are located on the short arm of their respective chromosomal group except *TaGGP2-A* which is located on the long arm. In addition to the six *TaGGP* genes, we identified two *HvGGP* genes within the barley (*Hordeum vulgare*) genome on chromosomes 4 and 5 (*HvGGP2* and *HvGGP1*, respectively), two *BdGGP* genes within the *Brachypodium distachyon* (*Brachypodium* hereafter) genome on chromosome 4 (*BdGGP1* and *BdGGP2*), two *AetGGP* genes within the *Aegilops tauschii* genome on chromosome 4 and 5 (*AetGGP2* and *AetGGP1,* respectively), and one *SbGGP* gene within the *Sorghum bicolor* L. genome on chromosome 8.

**Fig. 1:**
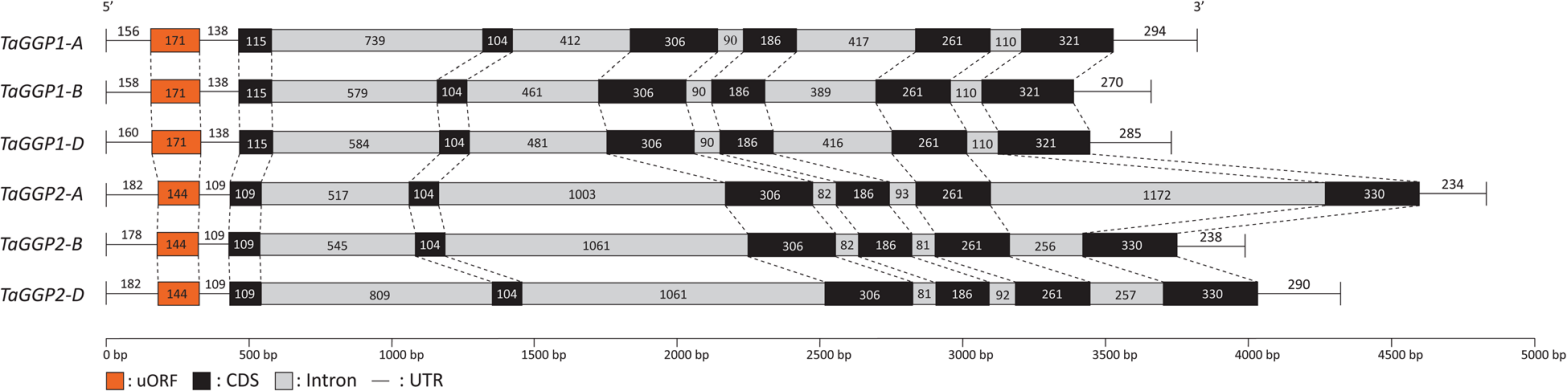
Gene structure of the *TaGGP* genes from bread wheat cv. Chinese Spring. The uORF (orange box), coding sequence (CDS, black box), introns (grey box), and UTR (lines) of the *TaGGP* genes are depicted and the length (bp) of each section indicated

**Fig. 2:**
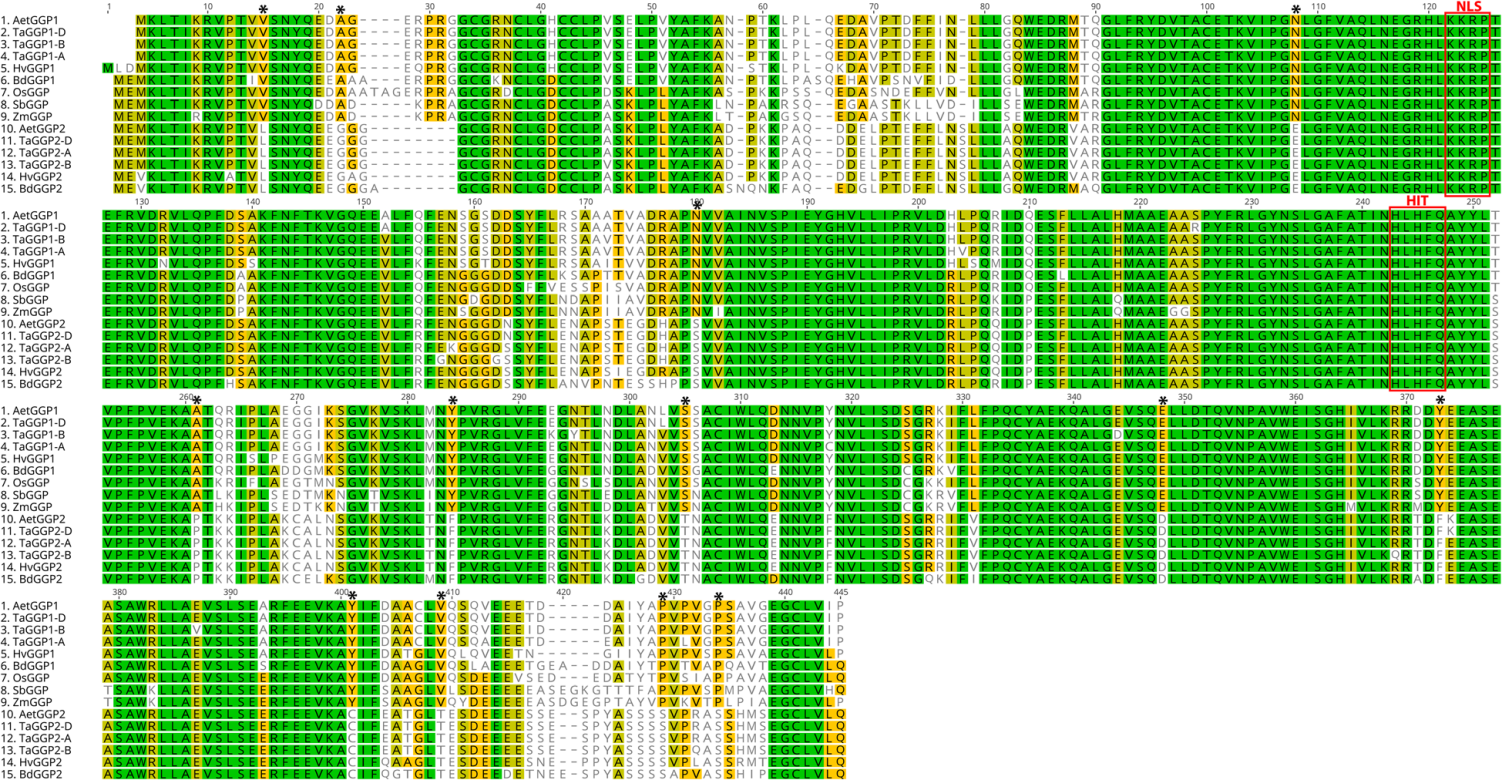
Amino acid sequence alignment of GGP proteins from a range of graminaceous species. Green, olive green, yellow and white background colour represents 100%, 80 to 100%, 60 to 80%, and less than 60% conservation of amino acids between species, respectively. The HIT motif (HφHφQ, where φ is a hydrophobic amino acid) of the HIT protein superfamily and the KKRP NLS are outlined in red. The 13 residues across the protein that distinguish GGP1 proteins from GGP2 proteins within the graminaceous species are indicated by an asterisk. The prefixes for the graminaceous species are as follows: Aet is *Aegilops tauschii*; Bd is *Brachypodium distachyon*; Hv is *Hordeum vulgare*; Os is *Oryza sativa*; Sb is *Sorghum bicolor*; Ta is *Triticum aestivum*; and Zm is *Zea mays*

The six *TaGGP* genomic sequences range in length from 2922 bp to 4163 bp mainly due to differences in intron sequence length (Fig. 1) and are similar in size and structure to other graminaceous *GGP* genes (Additional file 1: Figure S1). Genomic sequence comparisons of the six *TaGGP* genes revealed that they share between 36.0 to 94.9% identity. The *TaGGP1* and *TaGGP2* proteins are 430 and 431 amino acids in length, respectively, and sequence comparisons of the six *TaGGP* proteins revealed that they share between 78.1 to 99.3% identity. Alignment of the amino acid sequence of the wheat, barley, rice, maize, sorghum, *Brachypodium*, and *A. tauschii* GGP proteins identified highly conserved regions within the proteins, as well as the conserved histidine triad (HIT) motif of the HIT protein superfamily, the conserved KKRP nuclear localization signal (NLS), and 13 residues that distinguish GGP2 proteins from GGP1 proteins within these species (Fig. 2).

### Each *TaGGP* gene contains a highly conserved uORF

**Fig. 3:**
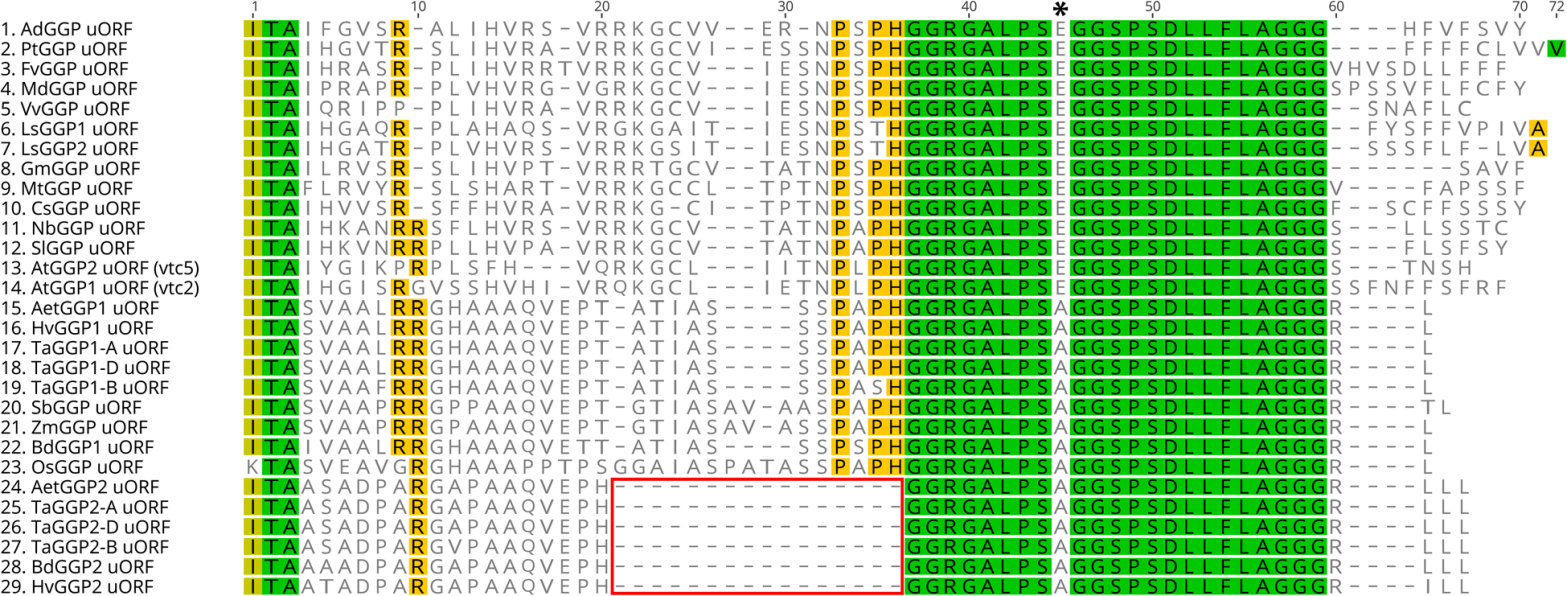
Amino acid sequence alignment of *GGP* uORFs from a range of graminaceous and non-graminaceous species. Green, olive green, yellow, and white background colour represents 100%, 80 to 100%, 60 to 80%, and less than 60% conservation of amino acids between species, respectively. The 45th residue of the consensus sequence distinguishing the graminaceous (A; alanine) from the non-graminaceous (E; glutamic acid) species is indicated with an asterisk. The truncated 11 residues from the *TaGGP2*, *HvGGP2*, *BdGGP2*, and *AetGGP2* uORF peptide sequences relative to the *TaGGP1*, *HvGGP1*, *BdGGP1*, and *AetGGP1* uORF peptide sequences are outlined in red. The prefixes for the graminaceous species are the same as those presented in Fig. 1. The prefixes for the non-graminaceous species are as follows: Ad is *Actinidia deliciosa*; At is *Arabidopsis thaliana;* Cs is *Cucumis sativus;* Fv is *Fragaria vesca*; Gm is *Glycine max*; Ls is *Lactuca sativa*; Md is *Malus x domestica*; Mt is *Medicago truncatula*; Nb is *Nicotiana benthamiana*; Pt is *Populus trichocarpa;* Sl is *Solanum lycospersicum*; and Vv is *Vitis vinifera*

For all six *TaGGP* genes, a highly conserved uORF was identified in the long 5’UTR of the mRNA initiating either from the non-canonical AUC (isoleucine) or ACG (threonine) start-codon. The uORF peptides of the *TaGGP1* homoeologs and *TaGGP2* homoeologs are 56 and 47 amino acids in length, respectively, and sequence comparisons of the six *TaGGP* uORF peptides revealed that they share between 67.9 to 100% identity. Alignment of the amino acid sequence of the *GGP* uORF peptides from a range of graminaceous and non-graminaceous species identified highly conserved regions within the peptides, as well as a lysine at the first residue in rice instead of a highly conserved isoleucine in all other species, a truncation of 11 residues in the *TaGGP2*, *HvGGP2*, *BdGGP2*, and *AetGGP2* uORF peptides relative to the *TaGGP1*, *HvGGP1*, *BdGGP1*, and *AetGGP1* uORF peptides, and a single amino acid change that distinguishes graminaceous species (alanine) from non-graminaceous species (glutamic acid) (Fig. 3).

### Graminaceous GGP proteins separate into two groups

**Fig. 4:**
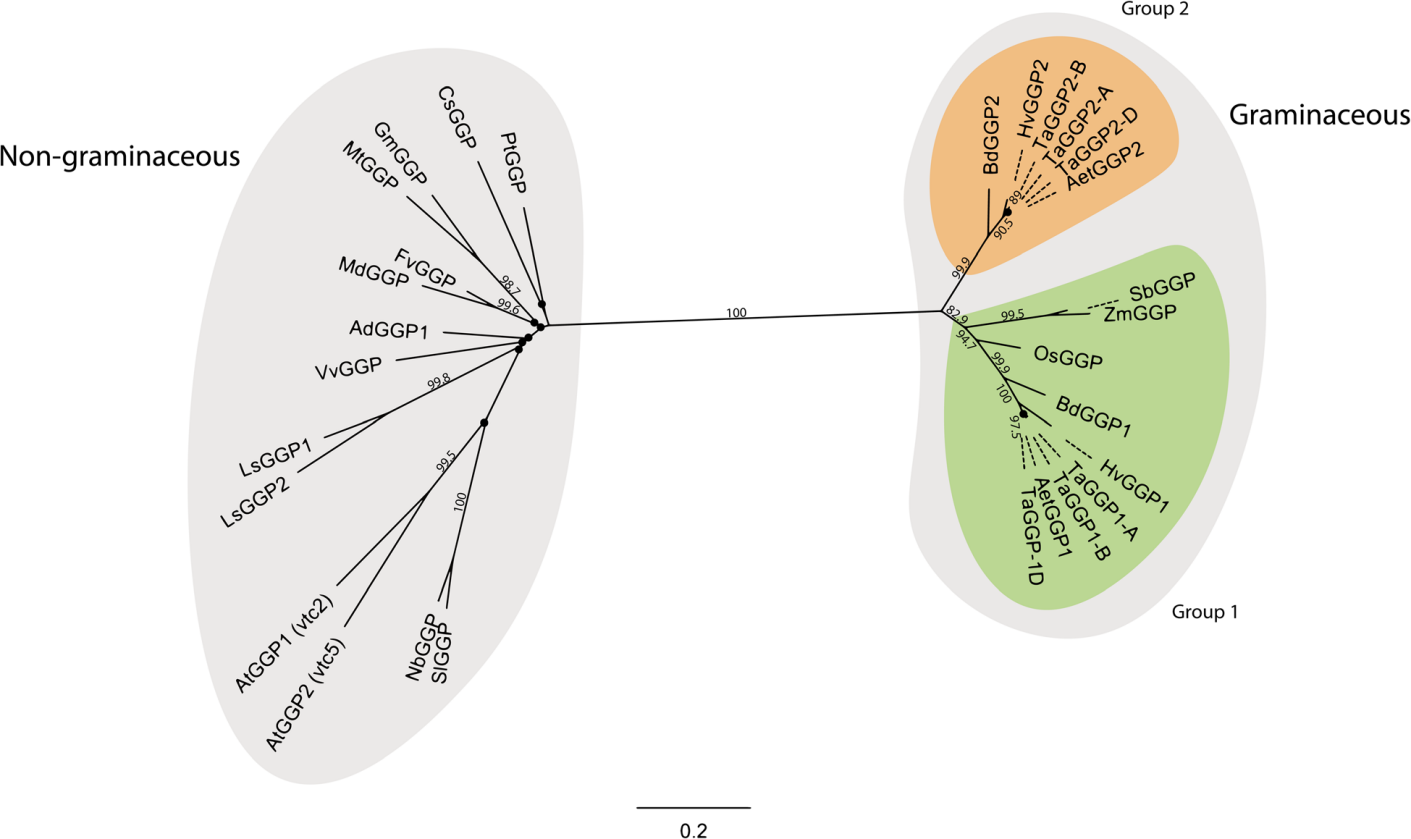
An unrooted phylogenetic tree of GGP proteins from a range of graminaceous and non-graminaceous species. Black nodes (●) represent weak bootstrap values (< 75%). The scale bar corresponds to evolutionary distance in substitutions per site and the numbers correspond to bootstrap percentage. The prefixes for the species are the same as those presented in Figs. 1 and 2

Phylogenetic analysis of the GGP protein sequences from a range of graminaceous and non-graminaceous plant species identified two clades of GGP proteins, belonging to either graminaceous or non-graminaceous species (Fig. 4). The graminaceous clade of GGP proteins further separates into two groups, with the TaGGP1 proteins forming one group with HvGGP1, BdGGP1, AetGGP1, OsGGP, ZmGGP, and SbGGP, and the TaGGP2 proteins forming a second group with HvGGP2, BdGGP2, and AetGGP2. This classification of group 1 and group 2 graminaceous GGP proteins is further supported by a phylogenetic analysis of the graminaceous *GGP* coding sequences which clearly demonstrates that the *TaGGP1* homoeologs, *HvGGP1*, *BdGGP1,* and *AetGGP1* are more closely related to the *OsGGP, ZmGGP,* and *SbGGP* in terms of evolutionary distance than the *TaGGP2* homoeologs, *HvGGP2*, *BdGGP2,* and *AetGGP2* (Additional file 1: Figure S2). Phylogenetic analysis of the *GGP* uORF peptide sequences revealed similar relationships to those observed for the GGP protein sequences (Additional file 1: Figure S3).

### The *TaGGP1* and *TaGGP2* subchromosomal regions do not share synteny

**Fig. 5:**
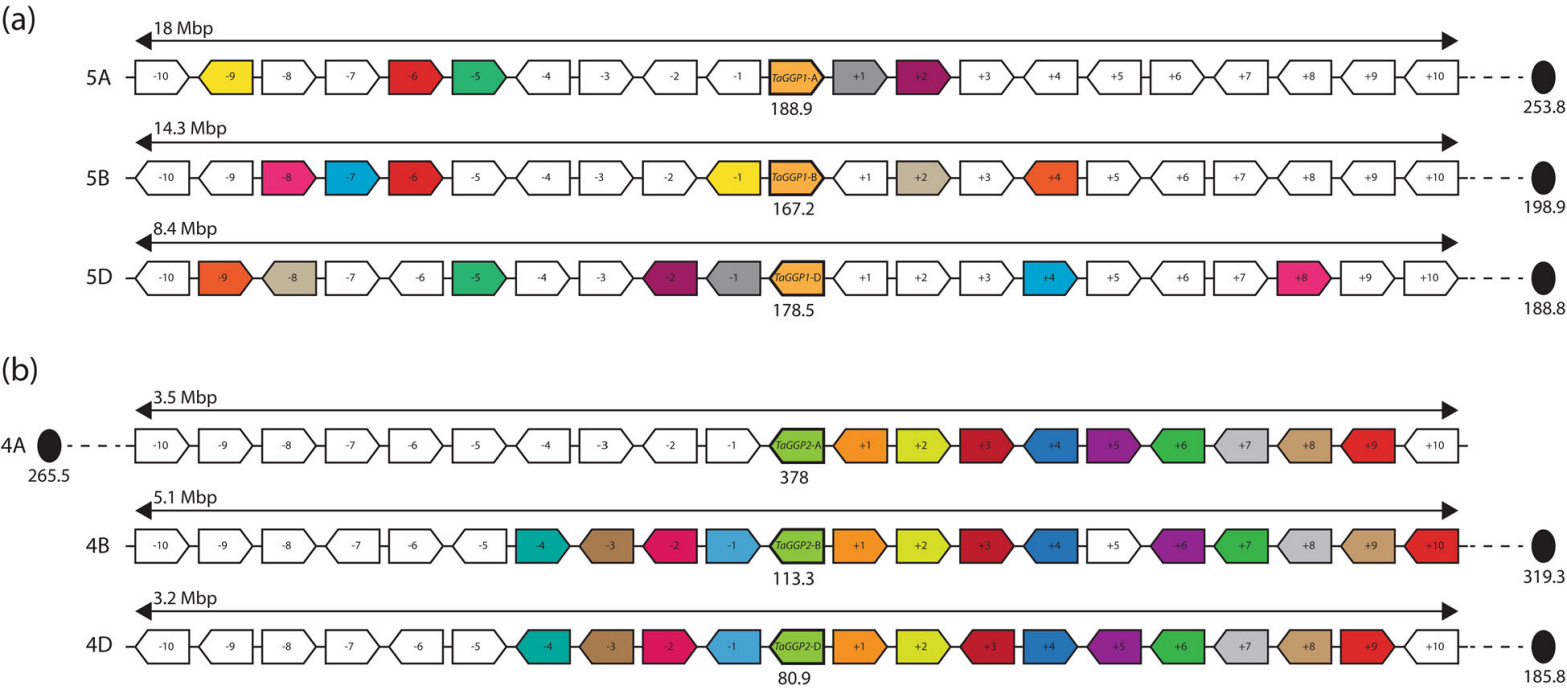
Microsynteny analysis of the *TaGGP* genes from bread wheat cv. Chinese Spring. (**a**) *TaGGP1* and (**b**) *TaGGP2* homoeologous SRs on chromosomal groups 5 and 4, respectively. Syntenic genes are represented by arrow colour. The orientation of the genes is indicated by the direction of the arrows. The physical length (Mbp) of each of the *TaGGP* SRs is provided for each chromosome. The physical position (Mbp) of the *TaGGP* genes and the centromeres (black oval) are also presented. The physical distance (Mbp) between the proximal gene of the SR and the respective centromere (black dotted line) is 58.2, 22.2 and 4.4 for the *TaGGP1-A*, *TaGGP1-B*, and *TaGGP1-D* SRs, respectively, and 127.5, 203.8 and 104.6 for the *TaGGP2-A*, *TaGGP2-B*, and *TaGGP2-D* SRs, respectively. The list of genes used in this analysis and their respective position and gene function are provided in Additional file 2: Table S1

Selection of ten genes upstream and downstream of the respective *TaGGP* genes on each of the six chromosomes, referred to as the *TaGGP* subchromosomal regions (SR), were analysed for gene density and synteny (Fig. 5 and Additional file 2: Table S1). The physical length of the *TaGGP1-A*, *TaGGP1-B,* and *TaGGP1-D* SRs were 18, 14.8, and 8.7 Mbp, respectively, and the *TaGGP2-A*, *TaGGP2-B,* and *TaGGP2-D* SRs were 3.4, 4.8, and 3 Mbp, respectively. No shared synteny was found between the *TaGGP1* and *TaGGP2* SRs on chromosomal groups 4 and 5 except for the *TaGGP* genes themselves. Synteny between the *TaGGP1* homoeologous SRs was much lower than that of the *TaGGP2* homoeologous SRs with only 19 genes having shared synteny—the *TaGGP1* homoeologs were the only genes to share synteny on all three homoeologous SRs—whereas the *TaGGP2* homoeologous SRs have 37 genes with shared synteny. The *TaGGP1* homoeologs are found in the centromeric region of their respective chromosomes, whereas the *TaGGP2* homoeologs are found in the interstitial region of their respective chromosomes between the pericentromeric and telomeric regions.

### The two groups of *TaGGP* genes have distinct expression patterns in bread wheat tissues

**Fig. 6:**
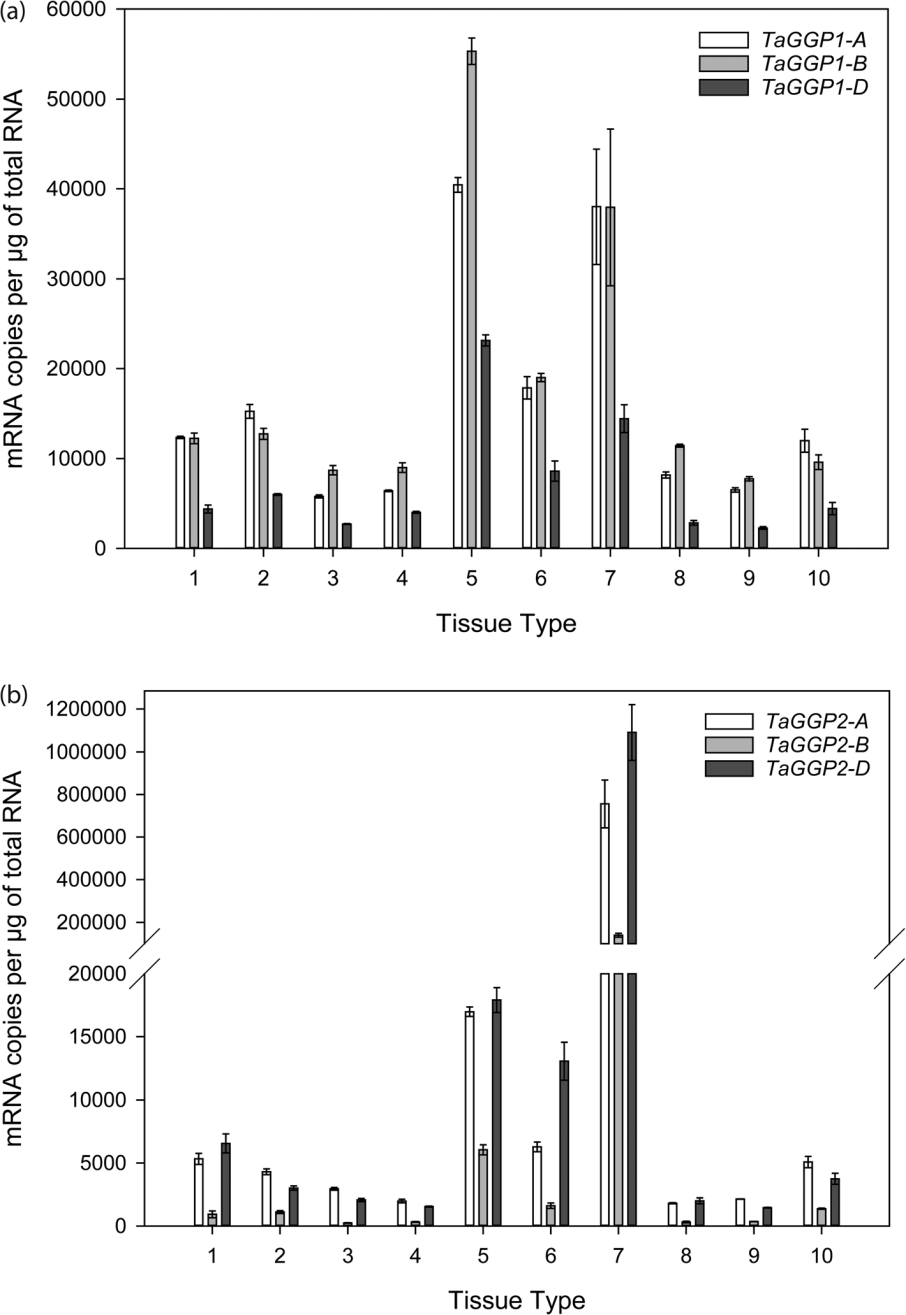
Quantitative reverse transcription-PCR analysis of the *TaGGP* genes from bread wheat cv. Chinese Spring. Relative expression of the (**a**) *TaGGP1* and (**b**) *TaGGP2* homoeologs is provided in: (1) embryonic root; (2) mesocotyl; (3) seedling root; (4) crown; (5) seedling leaf; (6) bracts; (7) anthers; (8) pistil; (9) caryopsis 3–5 DAP; and (10) embryo 22 DAP. The geometric mean of *TaActin*, *TaCyclophilin*, and *TaELF* were used as normalisation factors. Error bars indicate SEM of three technical replicates derived from a bulk of three independent biological samples

Quantitative reverse transcription-PCR analysis of a range of bread wheat tissues and developmental stages revealed distinct patterns of expression for the *TaGGP1* and *TaGGP2* genes (Fig. 6). The *TaGGP1* homoeologs were broadly expressed across different tissues and developmental stages, with the highest levels of expression detected in the seedling leaf, bracts, and anthers (Fig. 6a). The *TaGGP1* homoeologs displayed a trend of nonbalanced expression with the *TaGGP1-D* homoeolog having lower expression levels across all tissues relative to *TaGGP1-A* and *TaGGP1-B*. In contrast to the *TaGGP1* homoeologs, the *TaGGP2* homoeologs were highly expressed in the anthers, with approximately 50- to 580-fold higher expression in anthers relative to other tissues (Fig. 6b). The *TaGGP2* homoeologs also displayed a trend of nonbalanced expression with the *TaGGP2-B* homoeolog having lower expression levels across all tissues relative to *TaGGP2-A* and *TaGGP2-D*. Taken together, the *TaGGP* genes were most highly expressed in anthers, seedling leaf, and bracts and most lowly expressed in the crown, seedling root, and caryopses 3–5 DAP.

We searched for anther/pollen specific cis-regulatory elements in the 1-kb promoter region of the *TaGGP* genes to identify whether the high expression of the *TaGGP2* homoeologs in anthers could be explained due to differences in these regulatory elements (Additional file 1: Figure S4). However, the high level of expression of the *TaGGP2* homoeologs in anthers does not appear to correlate with the density of anther/pollen *cis*-regulatory elements surrounding the transcription start site (TSS), with a higher density of anther/pollen *cis*-regulatory elements instead being present in regions surrounding the TSS of the *TaGGP1* homoeologs (Additional file 1: Figure S4).

### Transient transformation of *TaGGP* genes in *N. benthamiana* leaf increases ascorbate concentration

**Fig. 7:**
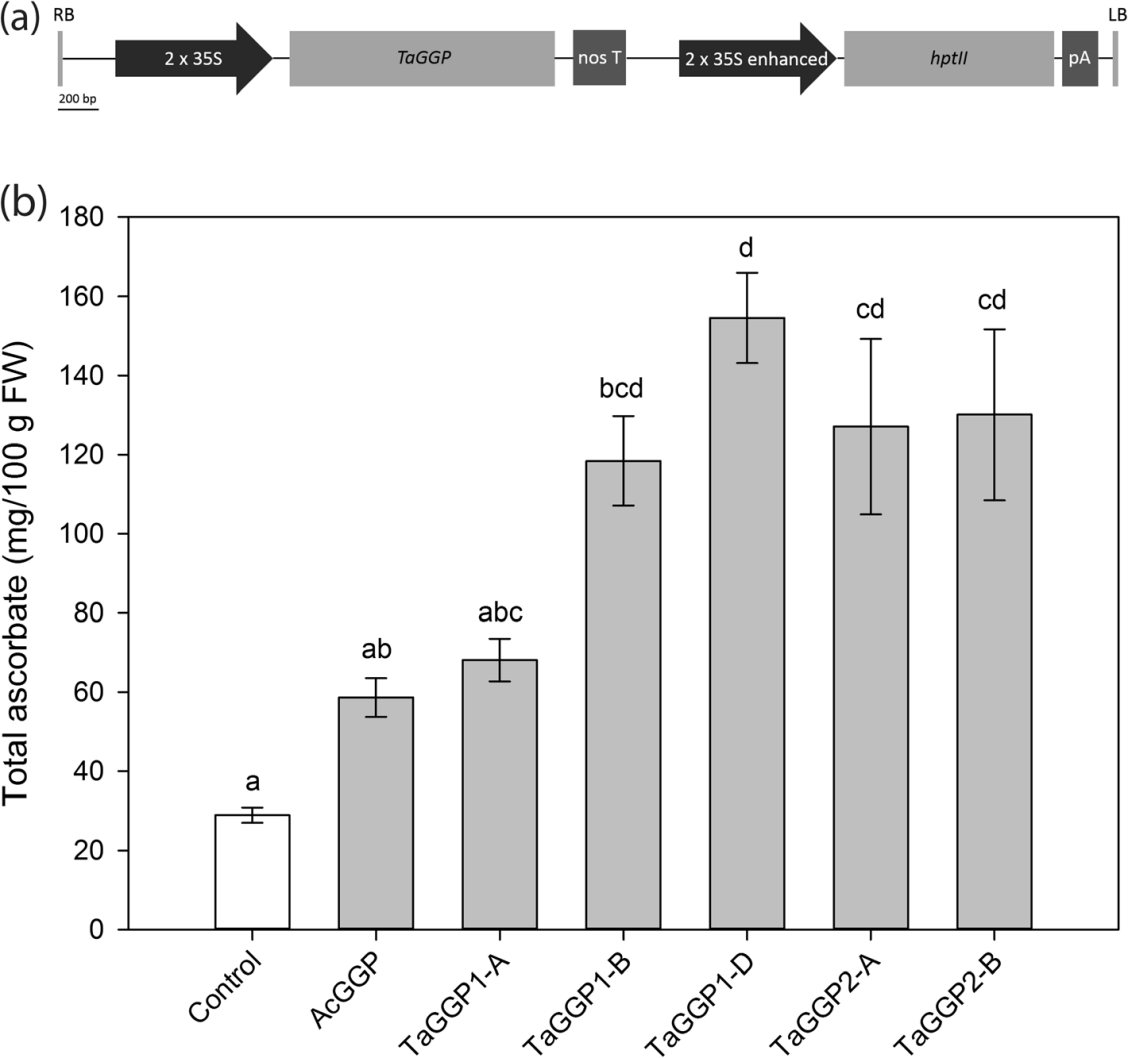
Transient transformation of the *TaGGP* coding sequences in *N. benthamiana*. **a** Schematic representation of the T-DNAs used for constitutive overexpression of the *TaGGP* genes in *N. benthamiana*. RB, right border; 2 x 35S, dual CaMV 35S promoter; *TaGGP*, coding sequence of *TaGGP1-A* (1,293 bp), *TaGGP1-B* (1,293 bp), *TaGGP1-D* (1,293 bp), *TaGGP2-A* (1,296 bp), and *TaGGP2-B* (1,296 bp); nos T, nopaline synthase terminator; 2 x 35S enhanced, dual CaMV 35S promoter enhanced; *hptII*, *hygromycin phosphotransferase II*; pA, CaMV poly(A) signal; LB, left border. **b** Ascorbate concentrations of *N. benthamiana* leaves co-infiltrated with *A. tumefaciens* (GV3101 MP90) containing the constructs of interest and a P19 construct to prevent post-transcriptional gene silencing. The control was infiltrated with *A. tumefaciens* containing the P19 construct alone. The *AcGGP* gene from kiwifruit driven by a single 35S promoter in the pGreen vector system was used as a positive control. Bars represent mean ± SEM of three infiltrated young leaves. Means that do not share a letter are significantly different (one-way ANOVA followed by Tukey post-hoc test with 95% confidence level)

Coding sequences of the *TaGGP1-A*, *TaGGP1-B*, *TaGGP1-D*, *TaGGP2-A*, and *TaGGP2-B* genes increased ascorbate concentrations in transiently transformed *N. benthamiana* leaf relative to the control (Fig. 7). Transformation with *TaGGP1-D* produced the greatest increase in ascorbate concentration (5.3-fold) relative to the control. Similar fold increases in ascorbate concentrations were also observed with transformation of *TaGGP1-B* (4.1-fold), *TaGGP2-A* (4.4-fold), and *TaGGP2-B* (4.5-fold). Transformation with *TaGGP1-A* produced a 2.4-fold increase relative to the control, which was the lowest increase in ascorbate concentration observed. *Agrobacterium tumefaciens* containing the *TaGGP2-D* construct were unable to be recovered. The higher fold increase in ascorbate observed for some of the *TaGGP* genes relative to the positive control *AcGGP* gene from kiwifruit may be explained by the strength of the promoters used to drive transgene expression: the dual 35S promoter used to drive the *TaGGP* genes is a stronger promoter than the single 35S promoter used to drive *AcGGP*.

## Discussion

### The *TaGGP* genes are highly conserved, functional, and have an uORF amenable to genome editing

We identified six *TaGGP* genes in the bread wheat genome, comprising of three *TaGGP1* homoeologs on chromosomal group 5 and three *TaGGP2* homoeologs on chromosomal group 4. Five of the genes are located on the short arm of their respective chromosomal group; the *TaGGP2-A* gene is located on the long arm of chromosome 4A due to a known pericentric inversion between the long and short chromosome arms [51]. We have also identified two copies of the *GGP* gene in the barley, *Brachypodium*, and *A. tauschii* genomes, as well as one copy in the sorghum genome. To our knowledge, this is the first time that the *HvGGP1*, *HvGGP2*, *BdGGP1*, *BdGGP2*, *AetGGP1*, *AetGGP2*, and *SbGGP* gene have been reported on.

The *TaGGP* protein sequences are highly conserved, particularly between homoeologs, with up to 99.3% shared identity (Fig. 2). This high level of conservation is striking given the genetic redundancy of the hexaploid wheat genome, which generally displays accelerated rates of coding sequence evolution relative to diploid organisms such as *Brachypodium* and suggests that all six *TaGGP* genes play important functional roles during wheat development and as such may be resistant to genetic drift [52]. This hypothesis is consistent with our finding that all five *TaGGP* genes transiently transformed into *N. benthamiana* leaf increased leaf ascorbate concentrations relative to the control, confirming their functional role in ascorbate biosynthesis *in planta* (Fig. 7). We can also conclude from the transient transformation that the 13 residues that distinguish the graminaceous GGP2 proteins from GGP1 proteins do not appear to affect the function of the GGP2 proteins. The substantial increase in ascorbate concentrations in *N. benthamiana* leaf observed for the *TaGGP* genes is consistent with GGP as the rate-limiting step towards ascorbate biosynthesis in plants [22, 24–26], and demonstrates the potential of utilising these genes, particularly *TaGGP1-D*, to increase ascorbate concentrations not only in wheat but other crops.

All six TaGGP proteins contained the highly conserved HφHφQ motif (where φ is a hydrophobic amino acid) that is characteristic of the galactose-1-phosphate uridylyltransferase branch of the HIT superfamily (Fig. 2.) [22, 23, 53]. They also all possess the highly conserved KKRP NLS, which may be responsible for localizing the *TaGGP* proteins to the nucleus, in addition to the cytoplasm where most of the ascorbate biosynthesis occurs (Fig. 2.) [54]. Fusion proteins of *AtGGP1*:YFP, *SlGGP1*-GFP, and *SlGGP2*:GFP have been found to localize to the cytoplasm and nucleus suggesting that GGP may be a dual-function protein serving both enzymatic and regulatory roles [54–56]. Determining the extent to which this NLS influences subcellular localization of GGP and what role GGP may be playing in the nucleus warrants further investigation.

We also identified a highly conserved uORF in the long 5’UTR of the mRNA for each of the six *TaGGP* genes (Figs. 1 and 3). Notably, the uORF of the *TaGGP2* homoeologs had a truncation of 11 residues relative to the *TaGGP1* homoeologs and the same truncation of 11 residues was also observed for the *BdGGP2* and *HvGGP2* uORFs relative to the *BdGGP1* and *HvGGP2* uORFs, respectively, indicating that this truncation occurred prior to the evolutionary divergence of *Brachypodium* and the Triticeae tribe (Fig. 3). If theses graminaceous *GGP2* uORFs are still functional this would suggest that these 11 residues are not critical to the functionality of the peptide. Disruption of the *GGP* uORF through genome editing has recently been shown as a viable strategy to increase ascorbate concentrations in a variety of plant species [34, 35]. For example, CRISPR/Cas9-targeted mutagenesis of the *AtGGP1* uORF in *Arabidopsis* and the *LsGGP1* and *LsGGP2* uORFs in lettuce to disrupt their function increased foliar ascorbate concentrations 1.7-, 1.4-, and 2.6-fold, respectively. Additionally, the increased ascorbate concentrations in lettuce conferred enhanced tolerance to methyl viologen-induced oxidative stress [35]. Similarly, CRISPR/Cas9-targeted mutagenesis of the *SlGGP2* uORF in tomato to disrupt its function increased foliar ascorbate concentrations approximately 1.4-fold [34]. Due to the plasticity of the hexaploid wheat genome, manipulating individual or multiple *TaGGP* uORFs with genome editing tools such as CRISPR/Cas9 represents a promising strategy to produce ascorbate-enriched wheat plants whilst also minimizing any pleiotropic effects on plant development that may arise due to disruption of the *GGP* uORF.

### A *Mutator*-like transposable element may be responsible for the duplication and specialized gene expression of *GGP2* in the *Brachypodium*/Triticeae lineage

We have categorized the graminaceous *GGP* genes described in this manuscript as group 1 or group 2 based on phylogeny. The protein and coding sequences of the *TaGGP1* homoeologs, *AetGGP1*, *HvGGP1*, and *BdGGP1* are more closely related to *OsGGP*, *ZmGGP*, and *SbGGP* in terms of evolutionary distance than that of the *TaGGP2* homoeologs, *AetGGP2*, *HvGGP2*, and *BdGGP2* (Fig. 4.). This grouping of graminaceous *GGP1* and *GGP2* genes is also consistent with the chromosomal location of the *GGP* genes and the known evolutionary history of grass genomes. For example, the wheat chromosomal group Ta5, barley chromosome Hv5, *Brachypodium* chromosome Bd4, rice chromosome Os12, and sorghum chromosome Sb8, which each harbour *GGP1* genes, are all descended from the ancestral grass chromosome A12 [57]. Whereas, the wheat chromosomal group Ta4, barley chromosome Hv4, and *Brachypodium* chromosome Bd4, which each harbour *GGP2* genes, are all descended from the ancestral grass chromosome A11 (in *Brachypodium* A11 and A12 were fused to form the single chromosome Bd4) [57]. Given that rice, maize, and sorghum each have a single copy of *GGP*, while wheat, barley, *Brachypodium*, and *A. tauschii* each have two copies of *GGP*, it appears that *GGP* may have been duplicated from A12 to A11 in the ancestor of the *Brachypodium*/Triticeae lineage. This duplication event would have occurred after the evolutionary divergence of the *Brachypodium*/Triticeae lineage from rice approximately 33.5 million years ago (MYA) but prior to the evolutionary divergence of *Brachypodium* and the Triticeae tribe approximately 24.5 MYA [57].

Our microsynteny analysis of the *TaGGP* genes revealed that the *TaGGP1* and *TaGGP2* SRs have no shared synteny except for the *TaGGP* genes themselves (Fig. 5.). We also did not find any shared synteny between the *BdGGP1* and *BdGGP2* SRs in *Brachypodium* (Additional file 2: Table S2) or between the *HvGGP1* and *HvGGP*2 SRs in barley (Additional file 2: Table S3). This lack of synteny between the *GGP1* and *GGP2* SRs in wheat, *Brachypodium*, and barley suggests that *GGP2* may have been duplicated via transposition in the ancestor of the *Brachypodium*/Triticeae lineage since other methods of gene duplication preserve gene synteny [58]. Given that the introns and gene structure of the wheat, *Brachypodium*, and barley *GGP2* genes have been preserved, a Class II transposable element (DNA transposon) that transposes via a DNA intermediate may have been responsible for such a transposition. We propose that a *Mutator*-like transposable element (MULE)—a family of DNA transposons that are prevalent in plants and capable of capturing, mobilizing, and amplifying gene fragments—may have been responsible for the duplication of *GGP2* [59]. However, given the proposed evolutionary time since *GGP2* was duplicated (24.5 to 33.5 MYA) any features of the transposable element would have degenerated by now and no longer be detectable [58].

We identified distinct expression patterns for the *TaGGP1* and *TaGGP2* genes with the *TaGGP1* homoeologs broadly expressed across different tissues and developmental stages, whereas the *TaGGP2* homoeologs were highly specialized with approximately 50- to 580-fold higher expression in anthers relative to other tissues (Fig. 6.). Our *TaGGP* gene expression results are supported by gene expression data extracted from http://bar.utoronto.ca/ which also demonstrates that the *TaGGP1* homoeologs are broadly expressed across different tissues and development stages, and that the *TaGGP2* homoeologs are highly expressed in anthers and leaves relative to other tissues (Additional file 1: Figure S5). Similar *GGP* expression profiles were also observed for other graminaceous species in terms of higher expression in anthers and/or leaves relative to other tissues (Additional file 1: Figures S5 and S6). To our knowledge this is the first time that specialized expression of a *GGP* gene in anthers has been documented, however, a previous study found high ascorbate concentrations correlated with high RNA concentrations in the developing anthers of jimsonweed (*Datura stramonium* L.) [60]. Together this expression data suggests that ascorbate may play an important role in anther development in wheat and other graminaceous species, for example protecting pollen from ROS induced by environmental stresses [61]. Determining whether the high expression of *GGP* in anthers is correlated with higher ascorbate concentrations relative to other tissues warrants further study.

In regards to the origin of the *cis*-regulatory elements responsible for the high expression of *GGP2* genes in the anthers relative to other tissues in wheat, *Brachypodium*, and barley, we propose two hypotheses: (i) following duplication, the regulatory region of the *GGP2* gene diverged due to a reduction in selection pressure, which subsequently generated de novo *cis*-regulatory elements, or (ii) *cis*-regulatory elements in the regulatory region were inherited from a transposable element that may have been responsible for the duplication of *GGP2.* In support of the first hypothesis, sequence alignments of the 1-kb promoter region of the wheat, *Brachypodium*, and barley *GGP* genes demonstrate that the promoter region of the *GGP2* genes have commonalities with the promoter region of the *GGP1* genes but have otherwise diverged significantly (Additional file 1: Figure S7). It is possible that sequence changes to the promoter region of *GGP2* genes have generated de novo *cis*-regulatory elements responsible for the high expression of *GGP2* genes in anthers. On the other hand, transposable elements are known to influence the expression of transposed genes [58]. For example, MULEs—including any sequences that have been captured, mobilized, and amplified by a MULE—have been shown to be preferentially expressed in reproductive tissues [58, 62]. The terminal inverted repeat (TIR) of MULEs are proposed to serve as the regulatory region for the expression of MULEs, including any transposed gene fragments [58, 62]. If *GGP2* was transposed by a MULE in the ancestor of the *Brachypodium*/Triticeae lineage, it is possible that *GGP2* inherited *cis*-regulatory elements responsible for the high expression of *GGP2* in anthers directly from the TIR of the MULE.

### Applications towards plant breeding

Abiotic stresses, such as high temperature, are responsible for major losses in global wheat productivity and are predicted to intensify due to climate change [63]. Identifying genetic strategies to mitigate or offset these losses will be essential for maintaining global wheat production levels. Exogenous application of ascorbate to wheat plants has been shown to alleviate salt- and drought-induced oxidative stress in many studies [64–69]. The development of high-ascorbate bread wheat cultivars through overexpression of the *TaGGP* genes or genome editing of one or more *TaGGP* uORFs to disrupt their function could help to mitigate wheat production losses caused by abiotic stress without the need for exogenous applications.

With respect to human nutrition, the development of bread wheat products with elevated levels of ascorbate may be limited due to the decline of ascorbate to negligible levels during grain maturation that may be difficult to overcome [70, 71]. However, wheat products made from germinated grain may provide a vehicle for ascorbate biofortification since germinated grain contains considerably higher levels of ascorbate relative to non-germinated grain [72, 73]. Germinated grain from bread wheat cultivars with an enhanced capacity to biosynthesize ascorbate could represent a novel way for improving human dietary intake of ascorbate.

## Conclusions

We identified six *TaGGP* genes in the bread wheat genome, each with a highly conserved uORF. Phylogenetic and microsynteny analyses revealed that *GGP2* may have been duplicated via a transposable element in the ancestor of the *Brachypodium*/Triticeae lineage. The *TaGGP2* homoeologs had specialized expression in anthers which may have been inherited from the TIR of a MULE. Transient transformation of the *TaGGP* coding sequences in *N. benthamiana* leaf increased ascorbate concentrations up 5.3-fold, confirming their activity *in planta*. We propose that the six *TaGGP* genes and uORFs identified in this study will provide a valuable genetic resource for increasing ascorbate biosynthesis in bread wheat to improve wheat abiotic stress tolerance and human nutrition.

## Methods

### Identification and naming of the *GGP* genes and uORFs

The *OsGGP* coding sequence (LOC4351698) was used as a query in BLASTN searches against the International Wheat Genome Sequencing Consortium (IWGSC) RefSeq v1.0 assembly of the wheat genome cv. Chinese Spring [48] to identify *TaGGP* coding sequences. Each *TaGGP* gene was assigned a unique name based on homoeologous grouping and their respective subgenomes and followed the recommended rules for gene symbolization in wheat (http://wheat.pw.usda.gov/ggpages/wgc/98/Intro.htm). Gene prediction models including predicted coding and proteins sequences were obtained from the IWGSC database. The *OsGGP* coding sequence was also used as a query in BLASTN searches against the genome sequence databases for barley (IBSC_v2), *Brachypodium* (Brachypodium_distachyon_v3.0), *A. tauschii* (Aet_v4.0) from https://plants.ensembl.org and for sorghum (Sorghum_bicolor_NCBIv3) from https://www.ncbi.nlm.nih.gov. The structure of the *GGP* genes was determined with Splign (https://www.ncbi.nlm.nih.gov/sutils/splign/splign.cgi) [74]. The *GGP* uORF coding and peptide sequences were manually identified in the 5’UTR of the *GGP* genes starting from the highly conserved AUC codon encoding isoleucine as previously described [33].

### Cloning and gene sequencing

Full-length gene sequences of *TaGGP1-A* and *TaGGP2-A* could not be retrieved from the IWGSC RefSeq v1.0 assembly of the wheat genome cv. Chinese Spring [48] due to each gene having a gap in intron 2 where sequence was missing. PCR primers were designed to amplify gene fragments spanning the gap of the *TaGGP1-A* and *TaGGP2-A* genes from cv. Chinese Spring genomic DNA using the respective IWGSC gene sequences and Primer3 software [75, 76]. The *TaGGP1* gene fragment was PCR amplified with forward primer 5′ - GGCAATCTGTTAGGCAAGCA − 3′ and reverse primer 5′ - TGTCAAAAACAGGTATCAGCAATTT - 3′. The *TaGGP2* gene fragment was PCR amplified with forward primer 5′ – AGTTCTTCCTAAATTCTCTCCTCCTT - 3′ and reverse primer 5′ – TTGAGGAATCACCTCACCCTA - 3′. PCR amplification cycles consisted of 1 cycle = 1 m 95 °C; 35 cycles = 20 s 95 °C, 30 s 61 °C, 30 s 72 °C; 1 cycle = 5 m 72 °C. PCRs were performed in a final volume of 20 μL for MyTaq™ HS Red DNA Polymerase (Bioline, MA, USA) according to manufacturer’s instructions. The PCR products were purified with DNA Clean & Concentrator-5 (Zymo Research, CA, USA) and cloned into the pGEM®-T Vector System (Promega, WI, USA) according to manufacturer’s instructions. Restriction enzyme digestion of the cloned PCR products in pGEM®-T with PvuII (New England Biolabs, MA, USA) according to the manufacturer instructions was used to discriminate the *TaGGP1-A* and *TaGGP2-A* gene fragments from their respective B and D homoeologous gene fragments prior to Sanger sequencing at the Australian Genome Research Facility Ltd. (http://www.agrf.org.au/).

### Sequence alignment and phylogenetic analysis of the GGP and uORF sequences

Sequence alignment and phylogenetic analysis of the *GGP* coding, protein, and uORF peptide sequences were performed in Geneious 10.2.2 using ClustalW alignment with default parameters and the PhyML Geneious plugin [77] with a K80 substitution model for nucleotide sequences or a LG substitution model for amino acid sequences and a bootstrap value of 1000. The *GGP* genes used in the sequence alignments and phylogenetic analyses are provided in Additional file 2: Table S4.

### Microsynteny analysis of the *TaGGP*, *BdGGP*, and *HvGGP* genes

Ten high confidence genes upstream and downstream of the six *TaGGP*, two *BdGGP*, and two *HvGGP* genes were retrieved from the IWGSC RefSeq v1.0 assembly of the wheat genome cv. Chinese Spring [48], the Brachypodium_distachyon_v3.0 assembly of the *Brachypodium* genome (https://plants.ensembl.org), and the IBSC_v2 assembly of the barley genome (https://plants.ensembl.org), respectively, excluding genes associated with retrotransposons and retrovirus related polyproteins, and used as query sequences in BLASTN searches against the respective databases with an E-value threshold of 1e^− 10^. The genes were considered syntenic based on their genomic location and sequence identity (≥90%). The list of the genes used in the microsynteny analysis of the six *TaGGP*, two *BdGGP*, and two *HvGGP* genes, and their respective position and gene function, are provided in Additional file 2: Tables S1, S2, and S3, respectively.

### Quantitative reverse transcription-PCR analysis of the *TaGGP* genes

The cDNA library of 10 different tissues and development stages of bread wheat cv. Chinese Spring was previously generated as described [78]. The quantitative reverse transcription-PCR analysis of the six *TaGGP* genes was carried out using subgenome specific PCR primer pairs (Additional file 2: Table S5) for each *TaGGP* gene as previously described [79]. Subgenome specificity of primer pairs was verified using cv. Chinese Spring nulli-tetrasomic DNA [80]. Primer efficiency was ≥97% for all primer pairs. A three gene normalisation factor of the most stable housekeeping genes *TaActin*, *TaCyclophilin*, and *TaELF* was used to normalise qRT-PCR expression data as previously described [78, 81]. Supplementary *GGP* gene expression data was extracted using the corresponding gene identifiers provided in Additional file 2: Table S4 from http://bar.utoronto.ca/ for wheat [82], barley [83], and maize [84], and from https://www.ebi.ac.uk for *Brachypodium*, rice, and sorghum [85].

### Analysis of *TaGGP* promoter regions

For each *TaGGP* gene, the first exon as well as the 1-kb upstream region, which was considered the promoter region, was extracted from the genome sequence databases for *T. aestivum* (IWGSC) from https://plants.ensembl.org. These sequences were used as a query in BLASTN searches against the NCBI expressed sequence tag (EST) database for *T. aestivum* (https://blast.ncbi.nlm.nih.gov) to identify *TaGGP* cap-trapped full-length cDNA sequences and the TTS. The identification of anther/pollen *cis*-acting elements was performed in Geneious 10.2.2 as previously described [79]. Sequence alignment of the 1-kb promoter region of the *GGP* genes was performed in Geneious 10.2.2 using the MAFFT Geneious plugin [86] with a L-INS-i algorithm.

### Transient transformation of *N. benthamiana* leaves

Bread wheat cv. Gladius was sourced from the Australian Grains Genebank (https://www.seedpartnership.org.au/associates/agg). PCR primers were designed to amplify the coding sequence of the *TaGGP1* homoeologs and *TaGGP2* homoeologs from bread wheat cv. Gladius cDNA using Primer3 software [75, 76]. The *TaGGP1* homoeologs were PCR amplified with forward primer 5′ – ATGAAGCTGACGATTAAGAGGGTA − 3′ and reverse primer 5′ – TCACGGAATTACGAGGCAG - 3′. The *TaGGP2* homoeologs were PCR amplified with forward primer 5′ – ATGGAGATGAAGCTGACGAT - 3′ and reverse primer 5′ – TCACTGAAGGACAAGGCAAC - 3′. PCR amplification cycles consisted of 1 cycle = 30 s 98 °C; 35 cycles = 10 s 98 °C, 15 s 61 °C, 45 s 72 °C; 1 cycle = 5 m 72 °C. PCRs were performed in a final volume of 50 μL for Phusion High-Fidelity DNA Polymerase (New England Biolabs) according to the manufacturer’s instructions. The PCR products were purified with DNA Clean & Concentrator-5 (Zymo Research), A overhangs added with MyTaq™ HS DNA Polymerase (Bioline), ligated into the pCR8/GW/TOPO vector system using the pCR™8/GW/TOPO® TA Cloning Kit (Invitrogen, CA, USA), and recombined into the pMDC32 vector system [87] using Gateway™ LR Clonase™ II Enzyme mix (Invitrogen) according to the manufacturer’s instructions. Cloning of the correct gene family member and gene orientation was confirmed by Sanger sequencing at the Australian Genome Research Facility Ltd. *A. tumefaciens* (GV3101 pMP90) containing the constructs of interest (except *TaGGP2*-D) were co-infiltrated with *A. tumefaciens* containing a P19 construct [88] to prevent post-transcriptional gene silencing into the younger leaves of 6-week-old *N. benthamiana* plants (LAB strain) grown in a plant growth room (16 h daylength, 24 °C) [89]. Infiltration conditions were conducted as previously described [90]. Infiltration with *A. tumefaciens* containing the P19 construct alone was used as a control. Infiltration with *A. tumefaciens* containing the kiwifruit *AcGGP* gene in the pGreen vector system [24, 91] was used as a positive control. Leaves were harvested for ascorbate measurements 7 d after infiltration and frozen on dry ice.

### Ascorbate measurement

Total ascorbate was extracted and measured as previously described with modifications [92]. Briefly, total ascorbate was extracted from ground, lyophilized tissue homogenised in extraction fluid containing 8% metaphosphoric acid, 2 mM EDTA, and 2 mM TCEP (Sigma-Aldrich, MO, USA) at 40 °C for 2 h. The extract was centrifuged and 5 μl of supernatant was injected onto a C18 3 μm 33 × 7 mm Alltima Rocket column (Hichrom Limited, UK) with a flow rate of 1 mL/m. The concentration was determined by reverse phase HPLC with UV detection at 245 nm on a Shimadzu (Japan) 8050 instrument (RT 1.43 m) using ascorbic acid as a standard (Sigma-Aldrich). The elution procedure was gradient with mobile phases A: MS grade water with 1% formic acid (Sigma-Aldrich) and B: MS grade acetonitrile with 1% formic acid (Sigma-Aldrich). The peak was validated as ascorbic acid by using L-ascorbic acid analytical standard (Sigma-Aldrich) with HPLC-MS Multi Reaction Monitoring (MRM) scan, qualifier transition m/z (+) = 177.05 > 95.00.

### Statistical analysis

Statistically significant differences between means were detected using a one-way ANOVA followed by a Tukey post-hoc test with a 95% confidence level using Minitab® 17.1.0 (http://www.minitab.com/en-us/).

## Supplementary information

**Additional file 1: Figure S1.** Gene structure of the *AetGGP*, *BdGGP*, *HvGGP*, *OsGGP*, *SbGGP*, and *ZmGGP* genes. **Figure S2.** An unrooted phylogenetic tree of *GGP* coding sequences from a range of graminaceous species. **Figure S3.** An unrooted phylogenetic tree of *GGP* uORF peptides from a range of graminaceous and non-graminaceous species. **Figure S4.** Annotation of anther/pollen *cis*-acting elements within the 1-kb promoter region of the *TaGGP* genes. **Figure S5.** *GGP* gene expression data extracted from http://bar.utoronto.ca/ for wheat and barley and https://www.ebi.ac.uk for *Brachypodium*. **Figure S6.** *GGP* gene expression data extracted from https://www.ebi.ac.uk for rice and sorghum and http://bar.utoronto.ca/ for maize. **Figure S7.** Nucleotide sequence alignment of the 1-kb promoter regions of wheat, *Brachypodium*, and barley *GGP* genes.

**Additional file 2: Table S1.** Ten high confidence genes upstream and downstream of the six *TaGGP* genes. **Table S2.** Ten high confidence genes upstream and downstream of the two *BdGGP* genes. **Table S3.** Ten high confidence genes upstream and downstream of the two *HvGGP* genes. **Table S4.** The *GGP* genes utilized in the manuscript for sequence alignments and phylogenetic analyses. **Table S5.** Primers used for the quantitative reverse transcription-PCR analysis of the six *TaGGP* and housekeeping genes.

## Data Availability

The *GGP* and uORF sequences, as well as the supplementary gene expression data, analysed during this study are available from public databases as outlined in the material and methods and supplementary material. The GenBank accession numbers for the bread wheat cv. Gladius *TaGGP* coding sequences generated during the current study are: *TaGGP1-A*, MK514258; *TaGGP1-B*, MK514259; *TaGGP1-D*, MK514260; *TaGGP2-A*, MK514261; *TaGGP2-B*, MK514262; and *TaGGP2-D*, MK514263. The GenBank accession numbers will be searchable after Feb 13, 2020 or once the accession numbers appear in print. The remaining datasets generated during the current study are available from the corresponding author on reasonable request.

## Abbreviations

5’UTR: 5′ untranslated region
EST: Expressed sequence tag
GGP: GDP-L-galactose phosphorylase
HIT: Histidine triad
mORF: Major open reading frame
MULE: *Mutator*-like transposable element
NLS: Nuclear localization signal
ROS: Reactive oxygen species
SR: Subchromosomal regions
TIR: Terminal inverted repeat
TSS: Transcription start site
uORF: Upstream open reading frame
