# Supplementary material for: Genome-wide identification and characterization of the GDP-L-galactose phosphorylase gene family in bread wheat
Source: BMC Plant Biol. 2019 Nov 26;19:515. doi: 10.1186/s12870-019-2123-1 (PMC6878703; doi:10.1186/s12870-019-2123-1)
Supplement: Supplementary file 1 — Additional file 1: Figure S1. Gene structure of the AetGGP, BdGGP, HvGGP, OsGGP, SbGGP, and ZmGGP genes. Figure S2. An unrooted phylogenetic tree of GGP coding sequences from a range of graminaceous species. Figure S3. An unrooted phylogenetic tree of GGP uORF peptides from a range of graminaceous and non-graminaceous species. Figure S4. Annotation of anther/pollen cis-acting elements within the 1-kb promoter region of the TaGGP genes. Figure S5. GGP gene expression data extracted from http://bar.utoronto.ca/ for wheat and barley and https://www.ebi.ac.uk for Brachypodium. Figure S6. GGP gene expression data extracted from https://www.ebi.ac.uk for rice and sorghum and http://bar.utoronto.ca/ for maize. Figure S7. Nucleotide sequence alignment of the 1-kb promoter regions of wheat, Brachypodium, and barley GGP genes. [file 12870_2019_2123_MOESM1_ESM.docx]

**
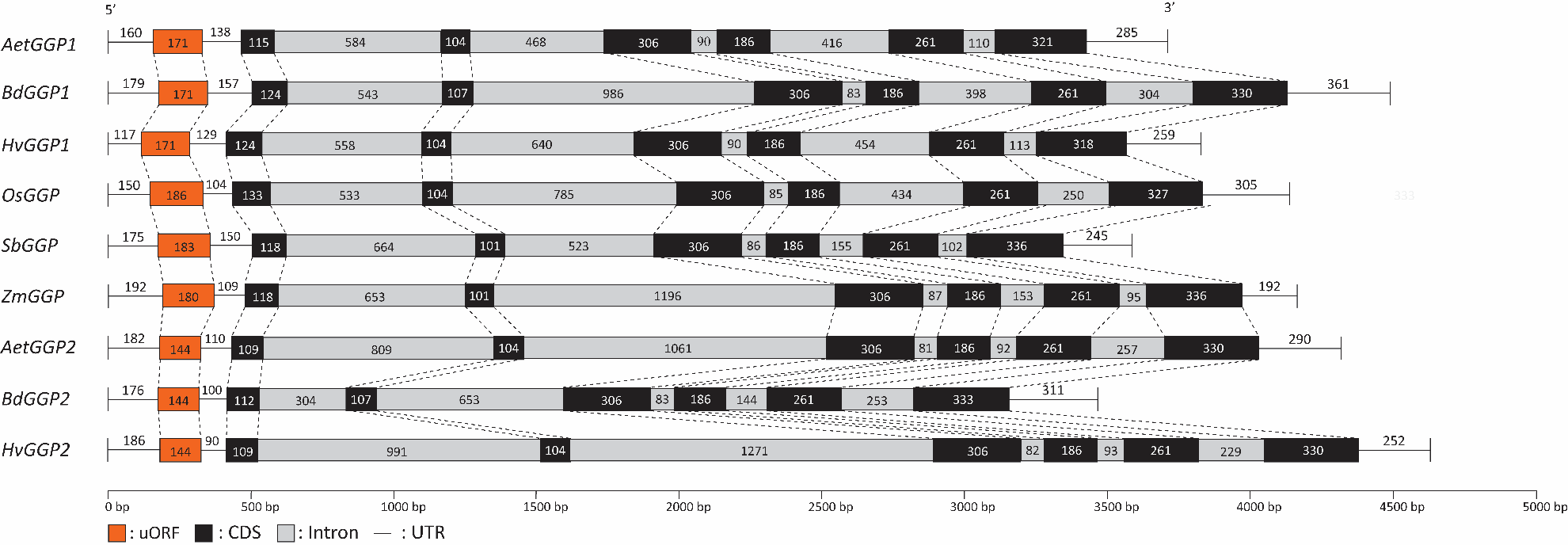
**

**Figure S1** Gene structure of the *AetGGP*, *BdGGP*, *HvGGP*, *OsGGP*, *SbGGP*, and *ZmGGP* genes. The uORF (orange box), coding sequence (CDS, black box), introns (grey box), and UTR (lines) of the *GGP* genes are depicted and the length (bp) of each section indicated. The prefixes for the graminaceous species are as follows: Aet is *Aegilops tauschii*; Bd is *Brachypodium distachyon*; Hv is *Hordeum vulgare*; Os is *Oryza sativa*; Sb is *Sorghum bicolor*; Ta is *Triticum aestivum*; and Zm is *Zea mays*.


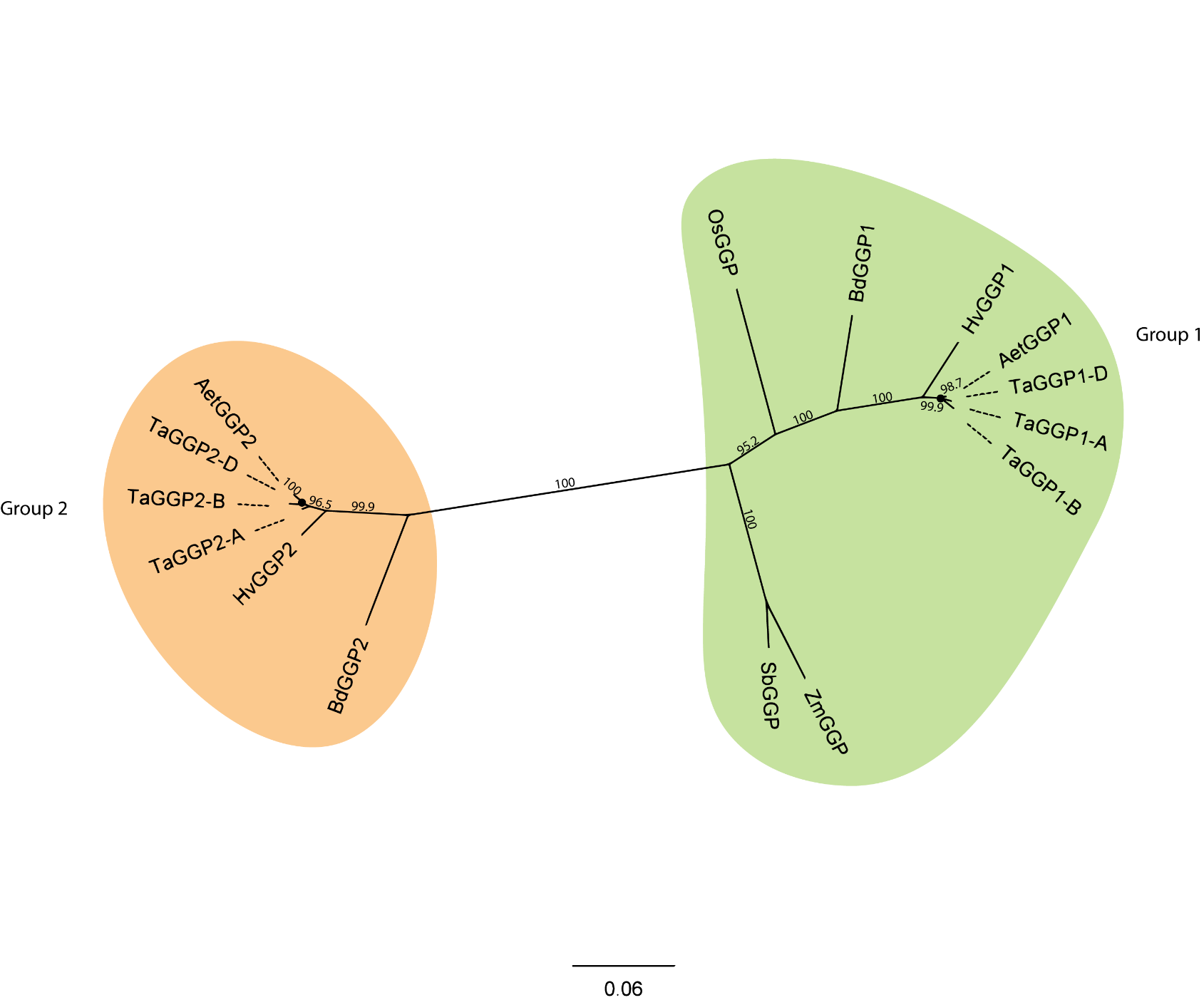


**Figure S2** An unrooted phylogenetic tree of *GGP* coding sequences from a range of graminaceous species. Black nodes (●) represent weak bootstrap values (<75%). The scale bar corresponds to evolutionary distance in substitutions per base and the numbers correspond to bootstrap percentage. The prefixes for the species are the same as those presented in Fig. 1 and 2.

**
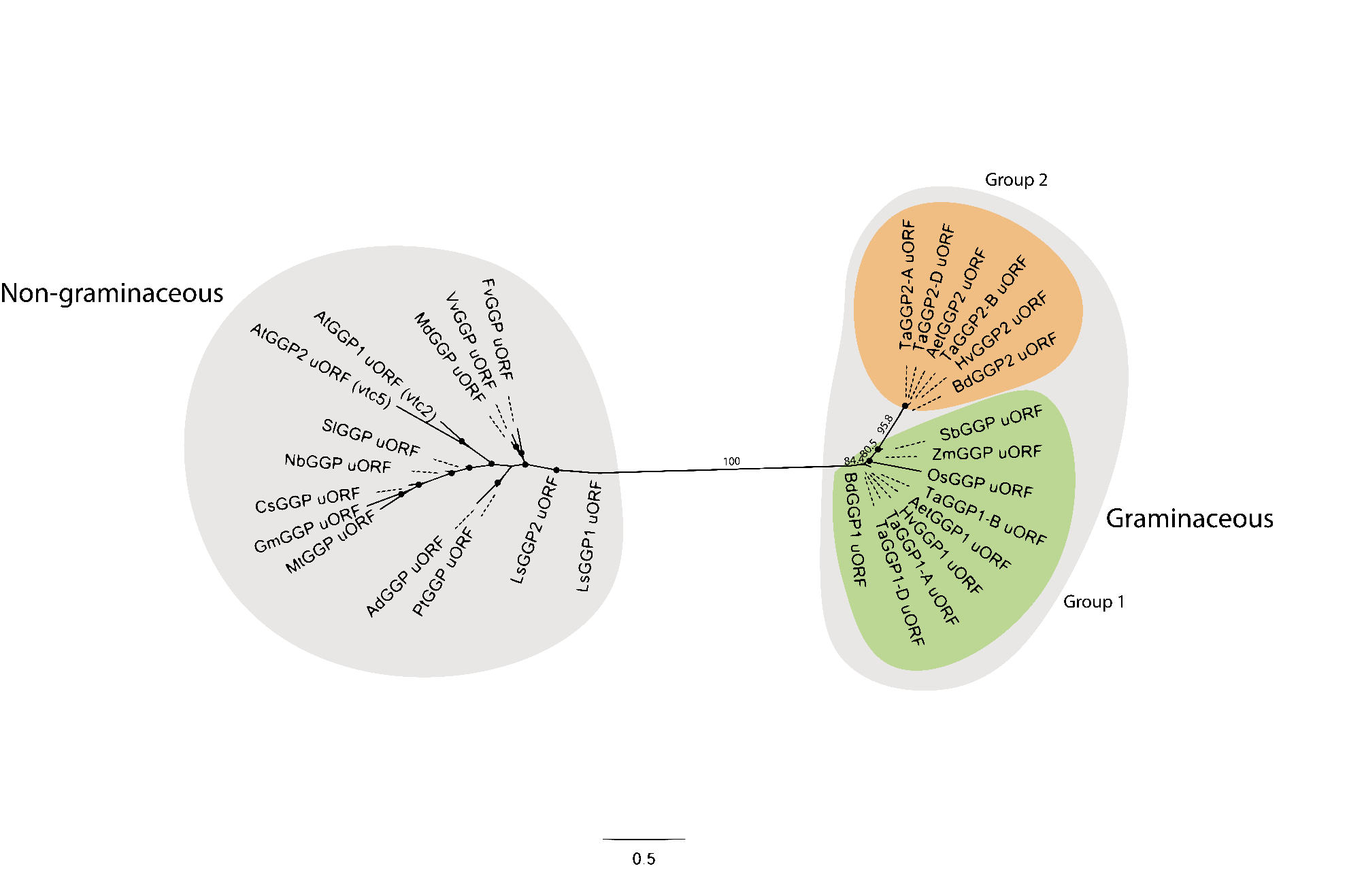
**

**Figure S3** An unrooted phylogenetic tree of *GGP* uORF peptides from a range of graminaceous and non-graminaceous species. Black nodes (●) represent weak bootstrap values (<75%). The scale bar corresponds to evolutionary distance in substitutions per site and the numbers correspond to bootstrap percentage. The prefixes for the species are the same as those presented in Fig. 1 and 2.


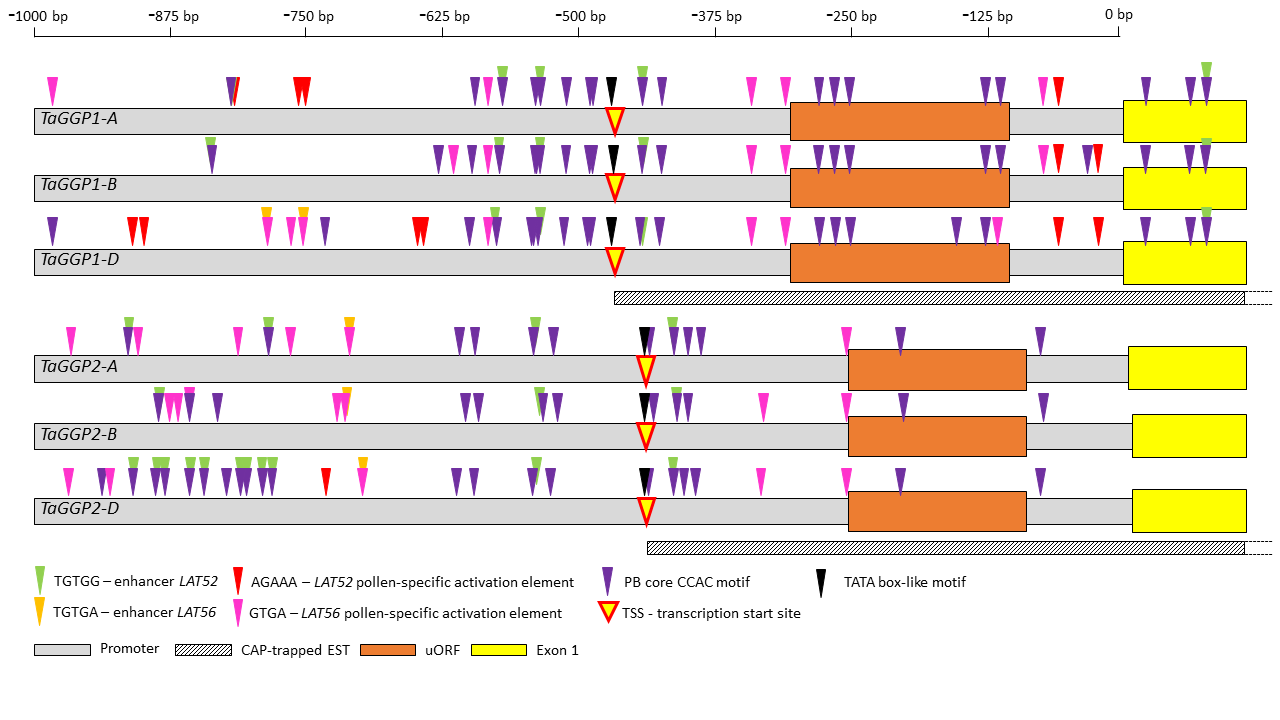


**Figure S4** Annotation of anther/pollen *cis*-acting elements within the 1-kb promoter region of the *TaGGP* genes. The promoter (grey), uORF (orange), and first exon (yellow) of the *TaGGP* genes are depicted. The CAP-trapped EST (striped) for the *TaGGP1* homoeologs and *TaGGP2* homoeologs are also depicted.


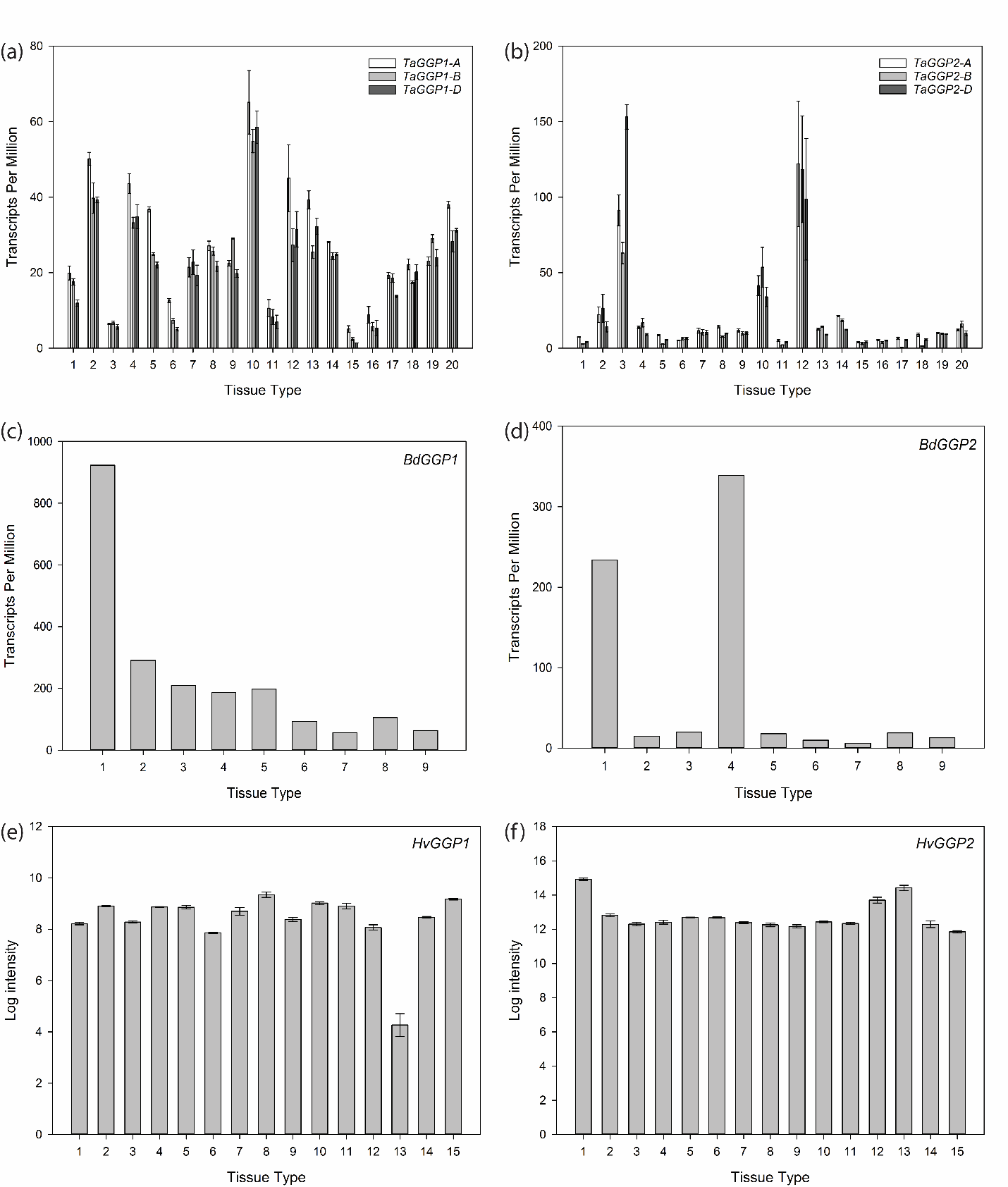


**Figure S5** *GGP* gene expression data extracted from <http://bar.utoronto.ca/> for wheat and barley and [https://www.ebi.ac.uk](https://www.ebi.ac.uk/gxa/experiments/) for *Brachypodium*. Relative expression of (**a**) *TaGGP1-A/B/D* (TraesCS5A01G107800, TraesCS5B01G111700, and TraesCS5D01G122900, respectively) and (**b**) *TaGGP2-A/B/D* (TraesCS4A01G201200, TraesCS4B01G105300 and TraesCS4D01G102200, respectively) is provided in: (1) grain; milk grain stage, (2) first leaf blade; seedling stage, (3) anther; anthesis, (4) coleoptile; seedling stage, (5) stigma and ovary; anthesis, (6) grain; ripening stage, (7) awns; ear emergence, (8) glumes; ear emergence, (9) flag leaf blade; 50 percent spike, (10) fifth leaf blade; flag leaf stage, (11) grain; soft dough, (12) flag leaf blade; flag leaf stage, (13) shoot axis; first leaf stage, (14) roots; seedling stage, (15) endosperm; dough stage, (16) grain; hard dough, (17) roots; tillering stage, (18) shoot axis; full boot, (19) fifth leaf blade; ear emergence, and (20) first leaf sheath; seedling stage. Error bars indicate SEM of three biological replicates. Relative expression of (**c**) *BdGGP1* (Bradi4g40740) and (**d**) *BdGGP2* (Bradi4g22700) is provided in: (1) leaves, (2) early inflorescence, (3) emerging inflorescence, (4) anther, (5) pistil, (6) seed 5 DAP, (7) seed 10 DAP, (8) embryo 25 DAP, and (9) endosperm 25 DAP. Relative expression of (**e**) *HvGGP1* (AK355342) and (**f**) *HvGGP2* (AK358028) is provided in: (1) leaf, (2) crown, (3) root, (4) coleoptile, (5) mesocotyl, (6) radicle, (7) caryopsis without embryo, (8) embryo 22 DAP, (9) caryopsis 5 DAP, (10) caryopsis 10 DAP, (11) caryopsis 16 DAP, (12) floral bracts, (13) anthers, (14) pistil, and (15) immature inflorescence. Error bars indicate SEM of three biological replicates.


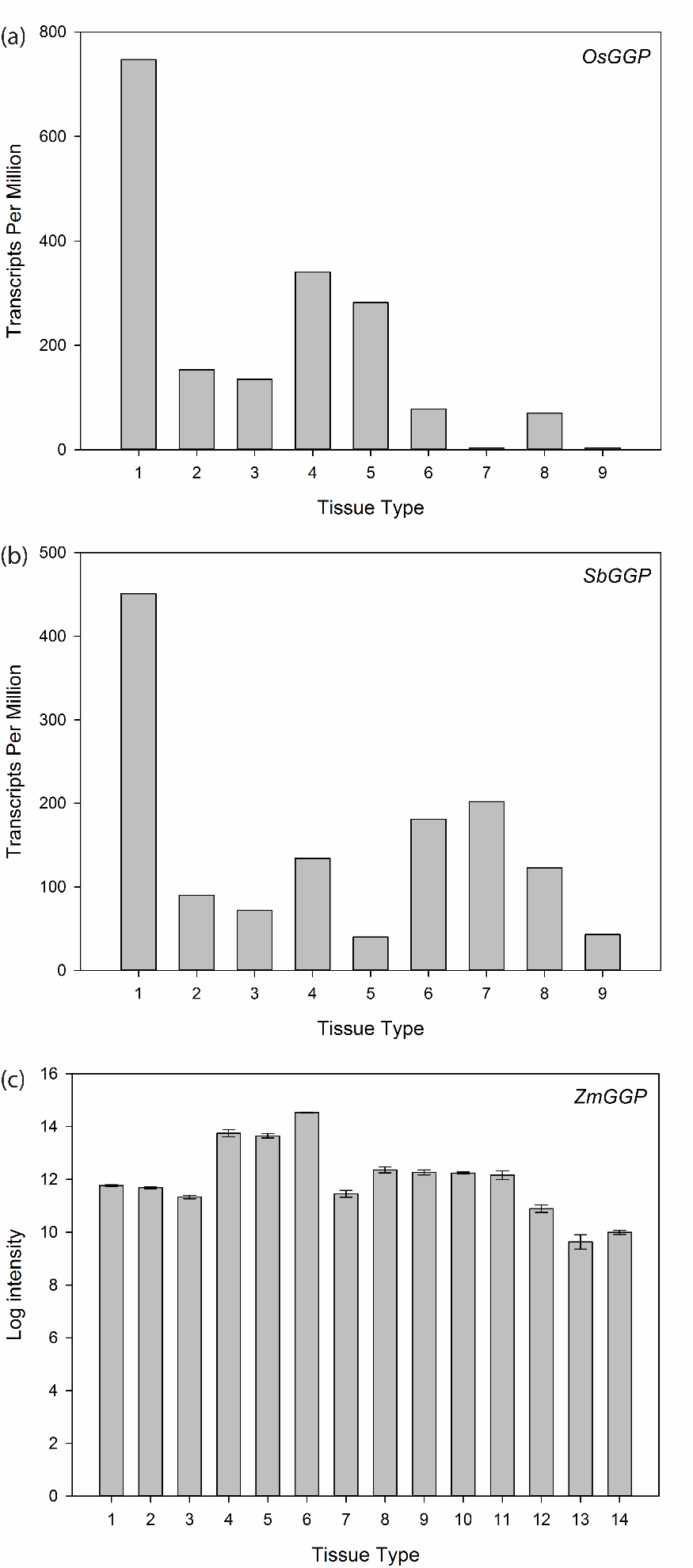


**Figure S6** *GGP* gene expression data extracted from https://www.ebi.ac.uk for rice and sorghum and http://bar.utoronto.ca/ for maize. Relative expression of (**a**) *OsGGP* (LOC_Os12g08810) and (**b**) *SbGGP* (SORBI_3008G064700) is provided in: (1) leaves, (2) early inflorescence, (3) emerging inflorescence, (4) anther, (5) pistil, (6) seed 5 DAP, (7) seed 10 DAP, (8) embryo 25 DAP, and (9) endosperm 25 DAP. Relative expression of (**c**) *ZmGGP* (GRMZM2G057186_T01) is provided in: (1) germinating seed 24 h, (2) coleoptile 6 DAS, (3) primary root 6 DAS, (4) anthers R1, (5) whole seedling VE, (6) pooled leaves V1, (7) pre-pollination cob R1, (8) silks R1, (9) embryo 16 DAP, (10) embryo 20 DAP, (11) embryo 24 DAP, (12) endosperm 16 DAP, (13) endosperm 20 DAP, and (14) endosperm 24 DAP. Error bars indicate SEM of three biological replicates.


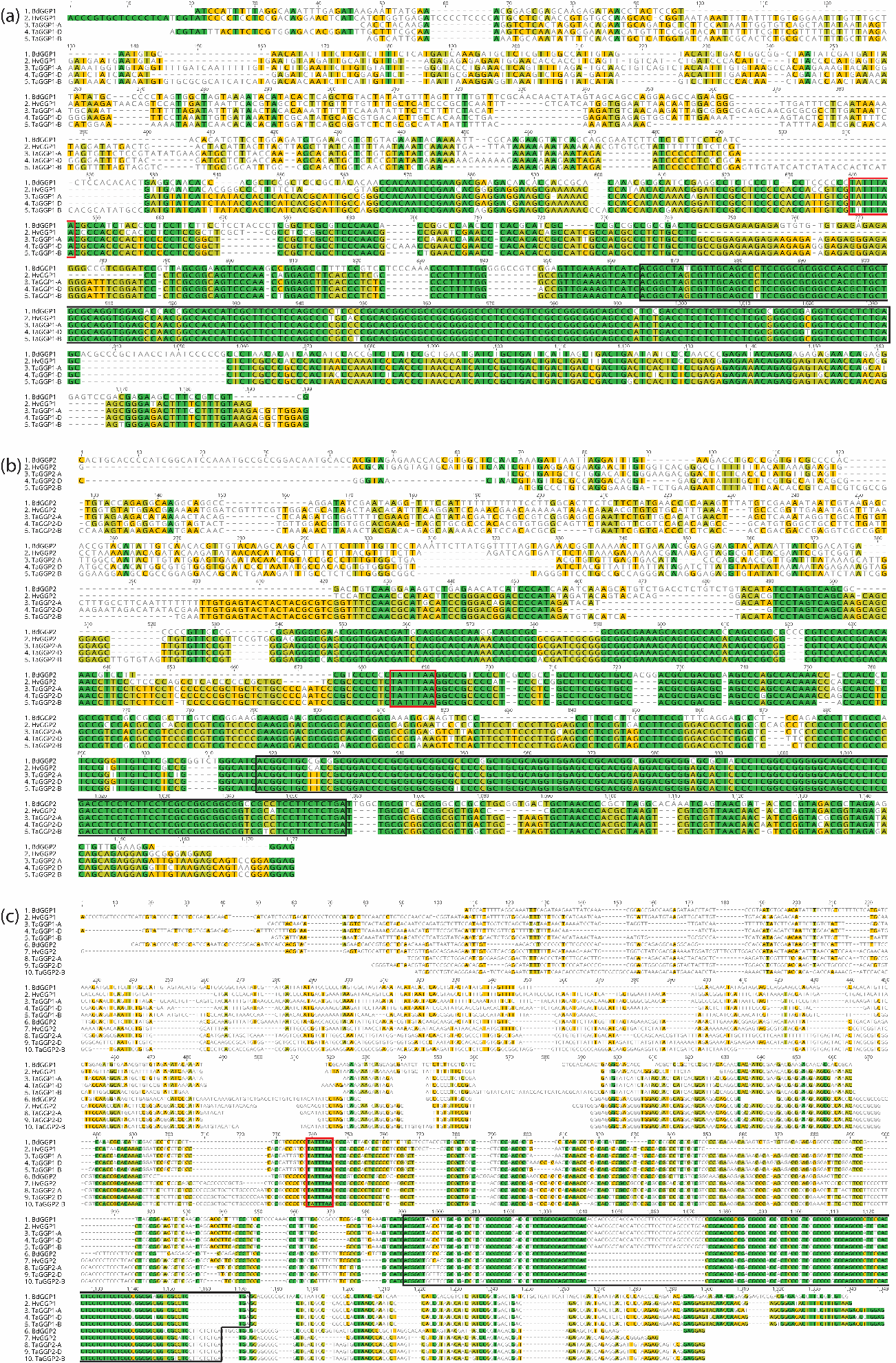


**Figure S7** Nucleotide sequence alignment of the 1-kb promoter regions of wheat, *Brachypodium*, and barley *GGP* genes. (**a**) *GGP1* genes, (**b**) *GGP2* genes, and (**c**) *GGP1* and *GGP2* genes. Green, olive green, yellow, and white background colour represents 100%, 80 to 100%, 60 to 80%, and less than 60% conservation of amino acids between species, respectively. The TATA box-like motifs and uORF are outlined in red and black, respectively.
