# Supplementary material for: Genome-wide identification and characterization of the GDP-L-galactose phosphorylase gene family in bread wheat
Source: BMC Plant Biol. 2019 Nov 26;19:515. doi: 10.1186/s12870-019-2123-1 (PMC6878703; doi:10.1186/s12870-019-2123-1)
Supplement: Supplementary file 2 — Additional file 2: Table S1. Ten high confidence genes upstream and downstream of the six TaGGP genes. Table S2. Ten high confidence genes upstream and downstream of the two BdGGP genes. Table S3. Ten high confidence genes upstream and downstream of the two HvGGP genes. Table S4. The GGP genes utilized in the manuscript for sequence alignments and phylogenetic analyses. Table S5. Primers used for the quantitative reverse transcription-PCR analysis of the six TaGGP and housekeeping genes. [file 12870_2019_2123_MOESM2_ESM.docx]

**Table S1** Ten high confidence genes upstream and downstream of the six *TaGGP* genes.

| **SR** | **Position** | **Gene ID** | **Gene Function** |
| --- | --- | --- | --- |
| ***TaGGP1-A*** | -10 | TraesCS5A01G106800 | Myb/SANT-like DNA-binding domain protein |
|  | -9 | TraesCS5A01G106900 | Protein transport protein Sec24-like |
|  | -8 | TraesCS5A01G107000 | Ribosomal RNA large subunit methyltransferase F |
|  | -7 | TraesCS5A01G107100 | kinesin 3 |
|  | -6 | TraesCS5A01G107200 | mediator of RNA polymerase II transcription subunit-like protein |
|  | -5 | TraesCS5A01G107300 | Protein kinase, putative |
|  | -4 | TraesCS5A01G107400 | uncharacterized |
|  | -3 | TraesCS5A01G107500 | Cell division topological specificity factor-like protein |
|  | -2 | TraesCS5A01G107600 | Transducin/WD40 repeat protein |
|  | -1 | TraesCS5A01G107700 | GRF1-interacting factor-like protein |
|  | 0 | TraesCS5A01G107800 | GDP-L-galactose phosphorylase 1 |
|  | 1 | TraesCS5A01G107900 | Calcium-dependent protein kinase |
|  | 2 | TraesCS5A01G108000 | Fructose-bisphosphate aldolase |
|  | 3 | TraesCS5A01G108100 | HSP20-like chaperones superfamily protein |
|  | 4 | TraesCS5A01G108200 | F-box/LRR-repeat protein 3 |
|  | 5 | TraesCS5A01G108300 | Isopentenyl-diphosphate delta-isomerase |
|  | 6 | TraesCS5A01G108400 | Chaperone protein DnaJ |
|  | 7 | TraesCS5A01G108500 | Cyclin family protein |
|  | 8 | TraesCS5A01G108600 | WWE protein-protein interaction domain protein family |
|  | 9 | TraesCS5A01G108700 | Nitrilase/cyanide hydratase and apolipoprotein N-acyltransferase family protein |
|  | 10 | TraesCS5A01G108800 | phragmoplast orienting kinesin 1 |
| ***TaGGP1-B*** | -10 | TraesCS5B01G110700 | AT-rich interactive domain-containing protein 2 |
|  | -9 | TraesCS5B01G110800 | Diacylglycerol kinase |
|  | -8 | TraesCS5B01G110900 | Methyltransferase |
|  | -7 | TraesCS5B01G111000 | Ubiquitin carboxyl-terminal hydrolase, putative |
|  | -6 | TraesCS5B01G111100 | mediator of RNA polymerase II transcription subunit-like protein |
|  | -5 | TraesCS5B01G111200 | Chaperone DnaK |
|  | -4 | TraesCS5B01G111300 | Exocyst complex component Sec10, putative, expressed |
|  | -3 | TraesCS5B01G111400 | Adenylate kinase |
|  | -2 | TraesCS5B01G111500 | RNA-directed DNA polymerase (Reverse transcriptase); Ribonuclease H |
|  | -1 | TraesCS5B01G111600 | Protein transport protein Sec24-like family |
|  | 0 | TraesCS5B01G111700 | GDP-L-galactose phosphorylase 1 |
|  | 1 | TraesCS5B01G111800 | Thioredoxin |
|  | 2 | TraesCS5B01G111900 | E3 ubiquitin-protein ligase RGLG2 |
|  | 3 | TraesCS5B01G112000 | SPFH/Band 7/PHB domain-containing membrane-associated protein family |
|  | 4 | TraesCS5B01G112100 | Cyclin T1 |
|  | 5 | TraesCS5B01G112200 | 30S ribosomal protein S4 |
|  | 6 | TraesCS5B01G112300 | Photosystem II reaction center PsbP family protein |
|  | 7 | TraesCS5B01G112400 | phospholipid:diacylglycerol acyltransferase |
|  | 8 | TraesCS5B01G112500 | ATP-dependent Clp protease ATP-binding subunit |
|  | 9 | TraesCS5B01G112600 | SGF29 tudor-like domain-containing protein |
|  | 10 | TraesCS5B01G112700 | Allantoinase |
| ***TaGGP1-D*** | -10 | TraesCS5D01G121800 | Nucleotide-binding protein PFLU_0879 |
|  | -9 | TraesCS5D01G121900 | Cyclin family protein |
|  | -8 | TraesCS5D01G122000 | E3 ubiquitin-protein ligase RGLG2 |
|  | -7 | TraesCS5D01G122200 | RNA-directed DNA polymerase (Reverse transcriptase) |
|  | -6 | TraesCS5D01G122300 | Aminopeptidase |
|  | -5 | TraesCS5D01G122400 | Protein kinase, putative |
|  | -4 | TraesCS5D01G122500 | DNA repair protein RadA |
|  | -3* | TraesCS5D01G122600 | Ribosome maturation factor RimP |
|  | -2* | TraesCS5D01G122700 | Fructose-bisphosphate aldolase |
|  | -1 | TraesCS5D01G122800 | Calcium-dependent protein kinase |
|  | 0 | TraesCS5D01G122900 | GDP-L-galactose phosphorylase 1 |
|  | 1 | TraesCS5D01G123000 | Protein reticulata-related 1, chloroplastic |
|  | 2 | TraesCS5D01G123100 | U-box domain-containing protein |
|  | 3 | TraesCS5D01G123200 | Quinolinate synthase A |
|  | 4 | TraesCS5D01G123300 | Ubiquitin carboxyl-terminal hydrolase, putative |
|  | 5 | TraesCS5D01G123400 | HAUS augmin-like complex subunit 6 |
|  | 6 | TraesCS5D01G123500 | Regulator of chromosome condensation (RCC1) family protein |
|  | 7 | TraesCS5D01G123600 | CCR4-NOT transcription complex subunit 11 |
|  | 8 | TraesCS5D01G123700 | Histone-lysine N-methyltransferase setd3 |
|  | 9 | TraesCS5D01G123800 | Katanin p60 atpase-containing subunit a1 |
|  | 10 | TraesCS5D01G123900 | tRNA-specific 2-thiouridylase MnmA |
| ***TaGGP2-A*** | -10 | TraesCS4A01G200200 | Two-component response regulator-like protein |
|  | -9 | TraesCS4A01G200300 | 5'-AMP-activated protein kinase beta-2 subunit protein |
|  | -8 | TraesCS4A01G200400 | Apoptosis-inducing factor |
|  | -7 | TraesCS4A01G200500 | Pentatricopeptide repeat-containing protein family |
|  | -6 | TraesCS4A01G200600 | Digalactosyldiacylglycerol synthase 1, chloroplastic |
|  | -5 | TraesCS4A01G200700 | Transcription factor-related family protein |
|  | -4 | TraesCS4A01G200800 | BEL1-like homeodomain protein 1 |
|  | -3 | TraesCS4A01G200900 | cysteine-rich RLK (RECEPTOR-like protein kinase) 40 |
|  | -2 | TraesCS4A01G201000 | Phox-associated domain,Phox-like,Sorting nexin isoform 3 |
|  | -1 | TraesCS4A01G201100 | B3 domain-containing protein |
|  | 0 | TraesCS4A01G201200 | GDP-L-galactose phosphorylase 2 |
|  | 1 | TraesCS4A01G201300 | Phosphatidylinositol 4-kinase |
|  | 2 | TraesCS4A01G201400 | Cysteine desulfurase |
|  | 3 | TraesCS4A01G201600 | E3 ubiquitin-protein ligase |
|  | 4 | TraesCS4A01G201700 | Peroxidase |
|  | 5 | TraesCS4A01G201800 | Receptor lectin kinase |
|  | 6 | TraesCS4A01G201900 | Fusarium resistance orphan protein |
|  | 7 | TraesCS4A01G202000 | Expressed protein-RZ53 |
|  | 8 | TraesCS4A01G202100 | Alcohol dehydrogenase |
|  | 9 | TraesCS4A01G202200 | Alcohol dehydrogenase |
|  | 10 | TraesCS4A01G202300 | Alcohol dehydrogenase |
| ***TaGGP2-B*** | -10 | TraesCS4B01G104300 | Transcriptional corepressor SEUSS |
|  | -9 | TraesCS4B01G104400 | Transcriptional corepressor SEUSS |
|  | -8 | TraesCS4B01G104500 | Endoglucanase |
|  | -7 | TraesCS4B01G104600 | Transcription factor, putative |
|  | -6 | TraesCS4B01G104700 | Ninja-family protein |
|  | -5 | TraesCS4B01G104800 | Exosome complex component RRP41 |
|  | -4 | TraesCS4B01G104900 | Kinase |
|  | -3 | TraesCS4B01G105000 | exosome complex exonuclease |
|  | -2 | TraesCS4B01G105100 | Heavy-metal-associated domain-containing protein, putative |
|  | -1 | TraesCS4B01G105200 | MYB transcription factor |
|  | 0 | TraesCS4B01G105300 | GDP-L-galactose phosphorylase 2 |
|  | 1 | TraesCS4B01G105400 | Phosphatidylinositol 4-kinase |
|  | 2 | TraesCS4B01G105600 | Cysteine desulfurase |
|  | 3 | TraesCS4B01G105700 | E3 ubiquitin-protein ligase |
|  | 4 | TraesCS4B01G105800 | Peroxidase |
|  | 5 | TraesCS4B01G105900 | SWI/SNF-related matrix-associated actin-dependent regulator of chromatin subfamily A-like protein 1 |
|  | 6 | TraesCS4B01G106000 | Protein kinase |
|  | 7 | TraesCS4B01G106100 | Fusarium resistance orphan protein |
|  | 8 | TraesCS4B01G106200 | Expressed protein-RZ53 |
|  | 9 | TraesCS4B01G106300 | Alcohol dehydrogenase |
|  | 10 | TraesCS4B01G106400 | Alcohol dehydrogenase |
| ***TaGGP2-D*** | -10 | TraesCS4D01G101100 | Coiled-coil domain-containing protein 18, putative isoform 1 |
|  | -9 | TraesCS4D01G101200 | Coiled-coil domain-containing protein 18, putative isoform 1 |
|  | -8 | TraesCS4D01G101400 | Myb/SANT-like DNA-binding domain protein |
|  | -7 | TraesCS4D01G101500 | Transcriptional corepressor SEUSS |
|  | -6 | TraesCS4D01G101600 | Premnaspirodiene oxygenase |
|  | -5 | TraesCS4D01G101700 | Transcriptional corepressor SEUSS |
|  | -4 | TraesCS4D01G101800 | protein kinase family protein |
|  | -3 | TraesCS4D01G101900 | exosome complex exonuclease |
|  | -2 | TraesCS4D01G102000 | Heavy-metal-associated domain-containing protein, putative, expressed |
|  | -1 | TraesCS4D01G102100 | MYB transcription factor |
|  | 0 | TraesCS4D01G102200 | GDP-L-galactose phosphorylase 2 |
|  | 1 | TraesCS4D01G102300 | Phosphatidylinositol 4-kinase |
|  | 2 | TraesCS4D01G102400 | Cysteine desulfurase |
|  | 3 | TraesCS4D01G102500 | E3 ubiquitin-protein ligase |
|  | 4 | TraesCS4D01G102600 | Peroxidase |
|  | 5 | TraesCS4D01G102700 | Kinase, putative |
|  | 6 | TraesCS4D01G102800 | Fusarium resistance orphan protein |
|  | 7 | TraesCS4D01G102900 | Expressed protein-RZ53 |
|  | 8 | TraesCS4D01G103000 | Alcohol dehydrogenase |
|  | 9 | TraesCS4D01G103100 | Alcohol dehydrogenase |
|  | 10 | TraesCS4D01G103200 | SWAP (Suppressor-of-White-APricot)/surp domain-containing protein |

*Both genes were detected as sense-antisense gene pair.

**Table S2** Ten high confidence genes upstream and downstream of the two *BdGGP* genes.

| **SR** | **Position** | **Gene ID** | **Gene Function** |
| --- | --- | --- | --- |
| ***BdGGP1*** | -10 | bradi4g40840 | unknown |
|  | -9 | bradi4g40830 | BTB/POZ domain-containing protein |
|  | -8 | bradi4g40810 | Phosphoribosyltransferase-like |
|  | -7 | bradi4g40800 | Thioredoxin M-type, chloroplastic |
|  | -6 | bradi4g40790 | Armadillo-type fold domain containing protein |
|  | -5 | bradi4g40785 | Tetratricopeptide repeat-like superfamily protein |
|  | -4 | bradi4g40780 | Photosystem I reaction centre subunit N |
|  | -3 | bradi4g40770 | YUCCA-like flavin monooxygenase |
|  | -2 | bradi4g40760 | TPR domain protein |
|  | -1 | bradi4g40750 | flavin-containing monooxygenase YUCCA10-like |
|  | 0 | bradi4g40740 | GDP-L-galactose phosphorylase 1 |
|  | 1 | bradi4g40727 | putative disease resistance protein |
|  | 2 | bradi4g40720 | growth regulator related protein |
|  | 3 | bradi4g40710 | photosystem II reaction center PsbP family protein |
|  | 4 | bradi4g40700 | cysteine desulfurase-like |
|  | 5 | bradi4g40690 | cysteine desulfurase-like |
|  | 6 | bradi4g40680 | Peroxidase superfamily protein |
|  | 7 | bradi4g40675 | Syntaxin-related protein KNOLLE (Syntaxin 111) |
|  | 8 | bradi4g40670 | Phosphomethylpyrimidine kinase type-1 domain containing protein |
|  | 9 | bradi4g40660 | BREVIS RADIX-like |
|  | 10 | bradi4g40647 | vignain-like |
| ***BdGGP2*** | -10 | bradi4g22777 | Transposase, IS4-like domain containing protein |
|  | -9 | bradi4g22770 | SEUSS transcriptional co-regulator |
|  | -8 | bradi4g22765 | uncharacterised |
|  | -7 | bradi4g22760 | STE_MEKK_ste11_MAP3K.20 |
|  | -6 | bradi4g22750 | unknown |
|  | -5 | bradi4g22745 | reverse transcriptase |
|  | -4 | bradi4g22740 | NB-ARC domain-containing protein |
|  | -3 | bradi4g22730 | MYB transcrition factor |
|  | -2 | bradi4g22715 | flavin-containing monooxygenase YUCCA10-like |
|  | -1 | bradi4g22707 | unknown |
|  | 0 | bradi4g22700 | GDP-L-galactose phosphorylase 2 |
|  | 1 | bradi4g22690 | phosphatidylinositol kinase |
|  | 2 | bradi4g22681 | No apical meristem protein domain containing protein |
|  | 3 | bradi4g22670 | NifS-like protein |
|  | 4 | bradi4g22660 | Peroxidase superfamily protein |
|  | 5 | bradi4g22658 | unknown function |
|  | 6 | bradi4g22656 | unknown function |
|  | 7 | bradi4g22653 | uncharacterised |
|  | 8 | bradi4g22651 | uncharacterised |
|  | 9 | bradi4g22650 | transposon protein |
|  | 10 | bradi4g22645 | uncharacterised |

**Table S3** Ten high confidence genes upstream and downstream of the two *HvGGP* genes.

| **SR** | **Position** | **Gene ID** | **Gene Function** |
| --- | --- | --- | --- |
| ***HvGGP1*** | -10 | HORVU5Hr1G032240 | uncharacterised |
|  | -9 | HORVU5Hr1G032230 | uncharacterised |
|  | -8 | HORVU5Hr1G032220 | uncharacterised |
|  | -7 | HORVU5Hr1G032210 | uncharacterised |
|  | -6 | HORVU5Hr1G032170 | Zn-Finger MYM-type protein like |
|  | -5 | HORVU5Hr1G032100 | uncharacterised |
|  | -4 | HORVU5Hr1G032050 | iron binding dioxygenoase |
|  | -3 | HORVU5Hr1G031990 | 2-oxoglutarate-dependent dioxygenase-like |
|  | -2 | HORVU5Hr1G031980 | ubiquitin carboxyl-terminal hydrolase |
|  | -1 | HORVU5Hr1G031970 | uncharacterised |
|  | 0 | HORVU5Hr1G031870 | GDP-L-galactose phosphorylase 1 |
|  | 1 | HORVU5Hr1G031770 | uncharacterised |
|  | 2 | HORVU5Hr1G031730 | uncharacterised |
|  | 3 | HORVU5Hr1G031720 | uncharacterised |
|  | 4 | HORVU5Hr1G031690 | cyclin-dependent kinase inhibitor |
|  | 5 | HORVU5Hr1G031650 | RNA-directed DDNA polymerase |
|  | 6 | HORVU5Hr1G031630 | 40S ribosomal protein S19 family |
|  | 7 | HORVU5Hr1G031600 | peroxisomal adenine nucleotide carrier |
|  | 8 | HORVU5Hr1G031590 | uncharacterised |
|  | 9 | HORVU5Hr1G031560 | neomenthol dehydrogenase-like |
|  | 10 | HORVU5Hr1G031550 | LIM domain containing protein |
| ***HvGGP2*** | -10 | HORVU4Hr1G017170 | SUPPRESSOR OF GAMMA RESPONSE 1-like |
|  | -9 | HORVU4Hr1G017160 | transcriptional corepressor SEUSS-like |
|  | -8 | HORVU4Hr1G017140 | transcriptional corepressor SEUSS-like |
|  | -7 | HORVU4Hr1G017110 | uncharacterised |
|  | -6 | HORVU4Hr1G017100 | STE_MEKK_ste11_MAP3K.3 - like |
|  | -5 | HORVU4Hr1G017090 | Serine Carboxypeptidase |
|  | -4 | HORVU4Hr1G017080 | interactor of constitutive active ROPs 2, chloroplastic |
|  | -3 | HORVU4Hr1G017070 | heavy metal-associated isoprenylated plant protein 23 |
|  | -2 | HORVU4Hr1G017060 | protein GPR107-like |
|  | -1 | HORVU4Hr1G017040 | MYB transcrition factor |
|  | 0 | HORVU4Hr1G017030 | GDP-L-galactose phosphorylase 2 |
|  | 1 | HORVU4Hr1G017010 | phosphatidylinositol 4-kinase beta 1-like |
|  | 2 | HORVU4Hr1G017000 | methyltransferases superfamily protein |
|  | 3 | HORVU4Hr1G016990 | uncharacterised |
|  | 4 | HORVU4Hr1G016980 | ATP-dependent zinc metalloprotease FTSH, chloroplastic |
|  | 5 | HORVU4Hr1G016970 | F-box domain containing protein |
|  | 6 | HORVU4Hr1G016950 | E3 ubiquitin-protein ligase |
|  | 7 | HORVU4Hr1G016940 | Peroxidase superfamily protein |
|  | 8 | HORVU4Hr1G016920 | uncharacterised |
|  | 9 | HORVU4Hr1G016880 | lectin protein kinase family protein |
|  | 10 | HORVU4Hr1G016860 | uncharacterised |

**Table S4** The *GGP* genes utilized in the manuscript for sequence alignments and phylogenetic analyses.

| **Gene Name** | **Gene Identifier** | **Gene Description** | **Source Website** |
| --- | --- | --- | --- |
| *AdGGP1* | Achn155031 | GDP-L-galactose phosphorylase | http://bioinfo.bti.cornell.edu |
| *AetGGP1* | LOC109780373 | GDP-L-galactose phosphorylase 2-like | https://www.ncbi.nlm.nih.gov |
| *AetGGP2* | LOC109741548 | GDP-L-galactose phosphorylase 2-like | https://www.ncbi.nlm.nih.gov |
| *AtGGP1 (vtc2)* | At4g26850 | GDP-L-galactose phosphorylase 1 | https://www.ncbi.nlm.nih.gov |
| *AtGGP2 (vtc5)* | At5g55120 | GDP-L-galactose phosphorylase VITAMIN C DEFECTIVE 5 (VTC5) | https://www.ncbi.nlm.nih.gov |
| *BdGGP1* | LOC100842186 | GDP-L-galactose phosphorylase 2 | https://www.ncbi.nlm.nih.gov |
| *BdGGP2* | LOC100843203 | GDP-L-galactose phosphorylase 2 | https://www.ncbi.nlm.nih.gov |
| *CsGGP* | LOC101205337 | GDP-L-galactose phosphorylase 1 | https://www.ncbi.nlm.nih.gov |
| *FvGGP* | LOC101314355 | GDP-L-galactose phosphorylase 2 | https://www.ncbi.nlm.nih.gov |
| *GmGGP* | LOC100800738 | GDP-L-galactose phosphorylase 1 | https://www.ncbi.nlm.nih.gov |
| *HvGGP1* | HORVU5Hr1G031870 | Predicted protein | [https://plants.ensembl.org](https://plants.ensembl.org/Hordeum_vulgare/Info/Index) |
| *HvGGP2* | HORVU4Hr1G017030 | Predicted protein | https://plants.ensembl.org |
| *LsGGP1* | LOC111899617 | GDP-L-galactose phosphorylase 2 | https://www.ncbi.nlm.nih.gov |
| *LsGGP2* | LOC111907963 | GDP-L-galactose phosphorylase 2-like | https://www.ncbi.nlm.nih.gov |
| *MdGGP* | LOC103427292 | GDP-L-galactose phosphorylase 2-like | https://www.ncbi.nlm.nih.gov |
| *MtGGP* | LOC11423375 | GDP-L-galactose phosphorylase 1 | https://www.ncbi.nlm.nih.gov |
| *NbGGP* | Niben101Scf08035g00004.1 | GDP-L-galactose phosphorylase 1 | [https://solgenomics.net](https://solgenomics.net/search/locus) |
| *OsGGP* | LOC4351698 | GDP-L-galactose phosphorylase 2 | https://www.ncbi.nlm.nih.gov |
| *PtGGP* | LOC7483618 | GDP-L-galactose phosphorylase 2 | https://www.ncbi.nlm.nih.gov |
| *SbGGP* | LOC8084736 | GDP-L-galactose phosphorylase 2 | https://www.ncbi.nlm.nih.gov |
| *SlGGP* | Solyc06g073320 | GDP-L-galactose hexose-1-phosphate guanylyltransferase | https://solgenomics.net |
| *VvGGP* | LOC100253859 | GDP-L-galactose phosphorylase 2 | https://www.ncbi.nlm.nih.gov |
| *ZmGGP* | LOC100283852 | VTC2 | https://www.ncbi.nlm.nih.gov |
| *TaGGP1-A* | TraesCS5A02G107800 | n/a | https://plants.ensembl.org |
| *TaGGP1-B* | TraesCS5B02G111700 | n/a | https://plants.ensembl.org |
| *TaGGP1-D* | TraesCS5D02G122900 | n/a | https://plants.ensembl.org |
| *TaGGP2-A* | TraesCS4A02G201200 | n/a | https://plants.ensembl.org |
| *TaGGP2-B* | TraesCS4B02G105300 | n/a | https://plants.ensembl.org |
| *TaGGP2-D* | TraesCS4D02G102200 | n/a | https://plants.ensembl.org |

**Table S5** Primers used for the quantitative reverse transcription-PCR analysis of the six *TaGGP* and housekeeping genes.

| **Gene name** | **Forward primer sequence (5’-3’)** | **Reverse primer sequence (5’-3’)** | **PCR product length (bp)** | **Annealing temperature (°C)** |
| --- | --- | --- | --- | --- |
| *TaGGP1-A* | GAAGCACGCTTTGAGGAAGT | CAGCTGAAGGGCCAACCA | 121 | 63 |
| *TaGGP1-B* | AAGCAGGCTCTGGGCGATG | GCTTCCGACAGAGAGACCAC | 164 | 63 |
| *TaGGP1-D* | TTGGGTGCCTTTGCAACTAT | GCAAGCACTGGAAACCAAA | 222 | 60 |
| *TaGGP2-A* | GTTCTCTTTCGCTTTGAGAAAGGT | CGGTCAAGGACACGTGGA | 155 | 65 |
| *TaGGP2-B* | AAGCATAAGTGGGAGGACAGA | ACGGTCAACACGGAACTCA | 156 | 66 |
| *TaGGP2-D* | AGGCTGCAAGCCCATACTTC | TTCCCTCTCTCAAACACCAAA | 220 | 60 |
| *TaActin* | GACAATGGAACCGGAATGGTC | GTGTGATGCCAGATTTTCTCCAT | 236 | 60 |
| *TaCyclophilin* | CAAGCCGCTGCACTACAAGG | AGGGGACGGTGCAGATGAA | 227 | 60 |
| *TaELF* | CAGATTGGCAACGGCTACG | CGGACAGCAAAACGACCAAG | 227 | 60 |
